# Global and regional prevalence of multimorbidity in the adult population in community settings: a systematic review and meta-analysis

**DOI:** 10.1016/j.eclinm.2023.101860

**Published:** 2023-02-16

**Authors:** Saifur Rahman Chowdhury, Dipak Chandra Das, Tachlima Chowdhury Sunna, Joseph Beyene, Ahmed Hossain

**Affiliations:** a Department of Public Health, North South University, Dhaka, Bangladesh; b Department of Health Research Methods, Evidence, and Impact (HEI), McMaster University, Hamilton, Ontario, Canada; c College of Health Sciences, University of Sharjah, Sharjah, United Arab Emirates; d Global Health Institute, North South University, Dhaka, Bangladesh

**Keywords:** Multimorbidity, Systematic review, Meta-analysis, Global prevalence, Chronic disease

## Abstract

**Background:**

Knowing the prevalence of multimorbidity among adults across continents is a crucial piece of information for achieving Sustainable Development Goal 3.4, which calls for reducing premature death due to non-communicable diseases. A high prevalence of multimorbidity indicates high mortality and increased healthcare utilization. We aimed to understand the prevalence of multimorbidity across WHO geographic regions among adults.

**Methods:**

We performed a systematic review and meta-analysis of surveys designed to estimate the prevalence of multimorbidity among adults in community settings. We searched PubMed, ScienceDirect, Embase and Google Scholar databases for studies published between January 1, 2000, and December 31, 2021. The random-effects model estimated the pooled proportion of multimorbidity in adults. Heterogeneity was quantified using I^2^ statistics. We performed subgroup analyses and sensitivity analyses based on continents, age, gender, multimorbidity definition, study periods and sample size. The study protocol was registered with PROSPERO (CRD42020150945).

**Findings:**

We analyzed data from 126 peer-reviewed studies that included nearly 15.4 million people (32.1% were male) with a weighted mean age of 56.94 years (standard deviation of 10.84 years) from 54 countries around the world. The overall global prevalence of multimorbidity was 37.2% (95% CI = 34.9–39.4%). South America (45.7%, 95% CI = 39.0–52.5) had the highest prevalence of multimorbidity, followed by North America (43.1%, 95% CI = 32.3–53.8%), Europe (39.2%, 95% CI = 33.2–45.2%), and Asia (35%, 95% CI = 31.4–38.5%). The subgroup study highlights that multimorbidity is more prevalent in females (39.4%, 95% CI = 36.4–42.4%) than males (32.8%, 95% CI = 30.0–35.6%). More than half of the adult population worldwide above 60 years of age had multimorbid conditions (51.0%, 95% CI = 44.1–58.0%). Multimorbidity has become increasingly prevalent in the last two decades, while the prevalence appears to have stayed stable in the recent decade among adults globally.

**Interpretation:**

The multimorbidity patterns by geographic regions, time, age, and gender suggest noticeable demographic and regional differences in the burden of multimorbidity. According to insights about prevalence among adults, priority is required for effective and integrative interventions for older adults from South America, Europe, and North America. A high prevalence of multimorbidity among adults from South America suggests immediate interventions are needed to reduce the burden of morbidity. Furthermore, the high prevalence trend in the last two decades indicates that the global burden of multimorbidity continues at the same pace. The low prevalence in Africa suggests that there may be many undiagnosed chronic illness patients in Africa.

**Funding:**

None.

**Research in context**

**Evidence before this study**

We searched PubMed, ScienceDirect, and Google Scholar for peer-reviewed papers and research reports on the prevalence of multimorbidity, using the search words 'prevalence' and 'multimorbidity' and similar terms published between January 1, 2000 and December 31, 2021. One meta-analysis combined 68 studies from 1992 to 2017 and showed that the global pooled prevalence of multimorbidity in community settings was 33.1%. In 2021, another meta-study focused on articles that investigated people in community settings from Latin America and the Caribbean.

**Added value of this study**

This research used studies until 2021 to analyze multimorbidity prevalence in community settings worldwide. South America has the highest prevalence of multimorbidity when comparing prevalence estimates across geographic regions. The prevalence difference was obtained across age groups, gender, country and income level, and study periods. For the first time in a subgroup study, we stratified the number of conditions to estimate the prevalence of multimorbidity. Studies that included mental health in the definition of multimorbidity resulted in a high pooled prevalence. Our research also uses statistical techniques to estimate the pooled prevalence of multimorbidity in adults while capturing heterogeneity in the estimates. This study summarizes the available evidence and encourages policymakers to use more standardized methods to reduce the burden of multimorbidity, which is a critical step toward meeting the sustainable development goal (SDG) goal of reducing premature mortality from non-communicable diseases by one-third through prevention and treatment by 2030.

**Implications of all the available evidence**

Our findings show that the landscape of multimorbidity prevalence has increased in the last two decades though it has remained relatively unchanged since 2010, implying a slow reduction in the burden of multimorbidity. About half of the South American adult population had multimorbidity, and thus these countries should take it as a priority agenda to develop more sustainable and integrated models of care. Research like this is crucial as the world tries to balance lowering the expense of multimorbidity on society and improving healthcare outcomes.

## Introduction

Multimorbidity has emerged as a significant public health issue in the world. It is typically defined as the presence of two or more chronic conditions at the same time in one individual.^1^ Multimorbidity has increased in various population groups due to population aging, lifestyle changes, improved socioeconomic conditions, and improved diagnostic capabilities by health services.^2^, ^3^, ^4^ Due to a lack of data from low-income countries and the use of different definitions of multimorbidity, a recent systematic review highlighted the need to estimate the prevalence of multimorbidity and patterns of multimorbidity.^5^

The high prevalence of multimorbidity has several negative consequences, including a high mortality rate, increased healthcare utilization, and increased healthcare expenses, influencing overall functioning and quality of life.^6^, ^7^, ^8^, ^9^, ^10^ According to a recent review and meta-analysis, those with at least two morbidities have a 1.73 times higher risk of death than people without multimorbidity.^8^ Moreover, healthcare demands and costs of multimorbidity continue to rise as populations age.^11^

Although few systematic reviews and meta-analyses on multimorbidity in community settings have been published in recent years, these included fewer studies or are restricted to a specific geographic region.^12^, ^13^, ^14^, ^15^ According to a systematic review and meta-analysis of studies with data collected between 1992 and 2017, the global pooled prevalence of multimorbidity in community settings was 33.1% (95% confidence interval: 30.0–36.3%).^12^ This prior study, however, did not look at how multimorbidity patterns changed over time or gave insight into multimorbidity definitions based on the number of conditions.

In recent years, many studies have been conducted to identify the clinical patterns of chronic conditions.^14^^,^^16^, ^17^, ^18^, ^19^ Two systematic reviews on multimorbidity identified depression, hypertension, and diabetes as the most prevalent co-occurring chronic diseases.^5^^,^^20^ Another study of multimorbidity identified cardiovascular and metabolic diseases as the most common diseases, followed by mental health disorders and musculoskeletal conditions.^21^ In a multi-national cross-sectional study of non-institutionalized adults aged 50 and over in Finland, Poland, Spain, China, Ghana, India, Mexico, Russia, and South Africa, hypertension, cataract, and arthritis were the most prevalent comorbid conditions.^22^ A study conducted in Germany among health-insured individuals aged 65 and older identified three broad multimorbidity patterns–cardiovascular/metabolic disorders, anxiety/depression disorders, and pain/neuropsychiatric disorders.^23^ It indicates that mental health disorders were prevalent in the studies, so we examined the prevalence of multimorbidity with and without mental health disorders.

These findings provide an explanation for the clinical patterns as well as the burden of multimorbidity that was observed among the studied people. An accurate and up-to-date prevalence estimation is critical to assess the impact of multimorbidity on public health and project effective and integrative interventions to reduce premature death due to multimorbidity. It is challenging to conduct a meta-analysis to estimate a global prevalence as the different studies used a different number of diseases and disease combinations. There is no gold standard for quantifying multimorbidity; definitions of multimorbidity and statistical approaches for evaluating prevalence differ greatly.^24^, ^25^, ^26^, ^27^, ^28^ But the trade-off of generating pooled estimate of multimorbidity exceed the drawbacks of the variability in the data. However, the prevalence of multimorbidity was not thoroughly assessed based on geographic regions, country's economic level, age, study periods, and the number of diseases considered for defining multimorbidity.

Given the growing concern about the rising burden of chronic diseases, understanding the prevalence of multimorbidity in the adult population is critical for developing preventive strategies. As a result, we conducted a systematic review and meta-analysis to examine the global and regional prevalence of multimorbidity and changes in multimorbidity prevalence over time among the adult population in community settings.

## Methods

### Search strategy

We searched PubMed, Google Scholar, Embase and ScienceDirect online databases to select peer-reviewed papers for our systematic review and meta-analysis. We screened observational studies (cross-sectional and baseline in a cohort) to determine the global prevalence of multimorbidity in the adult population in community settings. Our search included articles published in any language between January 2000 and December 2021, which would help minimize data heterogeneity and provide a more precise estimate of global multimorbidity prevalence. The screening was conducted primarily in English, but we also utilized the Google translation tool for article selection. A description of search terms is given in Appendix A. The search results were compiled using *Mendeley* citation management software. In addition to the database search, we explored references of selected studies and previously published systematic reviews on similar topics to incorporate all potential pertinent articles to construct our summary estimates. The Preferred Reporting Items for Systematic Review and Meta-analysis (PRISMA) checklist was followed in this study.^29^ The protocol was registered in the PROSPERO database (CRD42020150945).

### Selection criteria

Our systematic review included studies that (1) defined multimorbidity as having more than one underlying chronic conditions; (2) documented multimorbidity as the outcome of interest; (3) provided the number of participants in the study, with at least 200; (4) defined multimorbidity in the article, with at least five chronic conditions; (5) were observational studies, either cross-sectional or cohort, including adults 18 years and above; (6) published in years 2000–2021; and (7) were conducted in a community setting. Furthermore, only the recent study was considered if more than one study studied the same population. Only prevalence at baseline was included when the design was a cohort. Studies were excluded if they (1) focused only on comorbidity, (2) defined multimorbidity as more than two diseases (3) studied only inpatients or outpatients in hospital and primary care settings, (4) studied institutional population, i.e., people in nursing home, old home etc., (5) included acute conditions in the list of conditions, (6) used less than 5 conditions to define multimorbidity, or (7) were qualitative, interventional studies, opinion articles, conference presentations, books, letters, editorials, reviews, dissertations/theses, or abstracts.

### Data extraction and quality assessment

Using Covidence, two independent reviewers (S.R.C. and D.C.D.) screened the articles. The reviewers examined successively the titles, abstracts, and full texts of all possibly relevant articles identified by our searches. The differences in article selection and data extraction were handled by consensus and, if necessary, discussion with another reviewer (A.H.). Two independent reviewers (S.R.C. and T.C.S.) created a data-extraction form to establish the type of information to be extracted. The reviewers (S.R.C. and T.C.S.) recorded pertinent data on the name of the first author, study settings (e.g., country, year of publication, study period (start-end year), region), and study conduct (e.g., study design, population age and male percentage, number of study participants, data sources, method of ascertainment of morbidity, and minimum number of conditions included in multimorbidity), prevalence of multimorbidity, and number of participants with multimorbidity from the published article only. We further stratified the articles based on the country's income level (World Bank classification by income, GNI per capita).^30^ Moreover, the study participants were cross tabulated by age group and gender, and multimorbidity was documented whenever possible. If the prevalence of multimorbidity was not directly given, it was manually computed from the data supplied in the articles. In studies providing longitudinal prevalence estimates over a period, we utilized baseline prevalence. After settling any differences, the two reviewers (S.R.C. and T.C.S.) independently extracted the data, discussed the inputs, and revised the extracted data. Unresolved issues were resolved by involving a third reviewer (J.B.).

The Newcastle-Ottawa Scale (NOS), the tool for assessing the quality of non-randomized research, was used to determine the risk of bias for individual studies.^31^ The eight items of NOS are categorized into three domains of potential bias, namely “selection (representativeness of the sample, sample size, non-respondents, ascertainment of the exposure),” “comparability (the subjects in different outcome groups are comparable, based on the study design or analysis; and confounding factors are controlled),” and “outcome (assessment of the outcome and statistical test)”.^31^, ^32^, ^33^ A few points on the NOS were modified to be relevant to our research question (Supplementary File 1). The articles' methodological stringency, lucidity, and clarity are reflected in the subjective scores. However, we did not eliminate any articles based on their quality scoring. A study can be given one star for each item within the selection and outcome categories. For comparability, a maximum of two stars can be awarded. Thus, a cross-sectional study can be awarded a maximum of 10 stars (10 points), and a cohort study can be awarded a maximum of 9 stars (9 points). Overall, the studies were categorized as “low risk of bias (8–10 stars)”, “moderate risk of bias (6–7 stars)”, and “high risk of bias (0–5 stars)”. Two independent reviewers (S.R.C. and D.C.D.) assessed the quality of the included studies, and the discrepancies were resolved with discussion with the third reviewer (A.H.). The PRISMA statement consists of a 27-item checklist given in Supplementary File 2.

### Statistical analysis

The statistical analysis was performed using *meta* and *metafor* packages in the R statistical software (version 4.1.1). Multimorbidity prevalence was estimated as the ratio of the number of people with multimorbidity (numerator) and sample size (denominator). The numerator was derived from the percentage of people with multimorbidity when the numerator was not available. We obtained the pooled prevalence (with 95% CIs) of multimorbidity among the overall population from all studies and subgroups. The pooled prevalence was estimated using a random-effects model that allows the actual effect size to vary from study to study. The calculated proportion from each study and the combined effect estimate with 95% CI were represented graphically using forest plots. We assessed potential publication bias by visually observing the symmetry of funnel plots and using Egger's test. The *I*^*2*^ statistic was used to quantify heterogeneity across the selected studies. The *I*^*2*^ statistic indicates the proportion of overall variation across studies due to heterogeneity rather than chance. Subgroup analysis was carried out to determine the pooled prevalence for each group and look for potential explanations for the heterogeneity. Geographical region (Africa, Asia, Europe, North America, Oceania, and South America); WB/WHO income region (High, Upper-middle, Low- and Lower-middle); Study design (Cross-sectional, Cohort); Multimorbidity (5–9 conditions, 10–19 conditions, ≥20 conditions); Mental health included in the multimorbidity definition (Yes or No); Age groups of study participants (≥30 years, ≥40 years, ≥50 years, ≥60 years) and Gender (male and female) were considered for sub-group analysis. We conducted a trend analysis to see the global multimorbidity prevalence over time (2000–2021). We also conducted sensitivity analyses to assess the findings' robustness in consideration of sample size, multimorbidity prevalence, multimorbidity definitions based on the number of conditions studied, and NOS overall quality of the studies. Two-sided P < .05 was considered statistically significant.

### Role of the funding source

There was no funding available for this study. All of the study's data was accessible to all of the authors, and the corresponding author had responsibility for publication.

## Results

### Identification and selection of studies

**Fig. 1:**
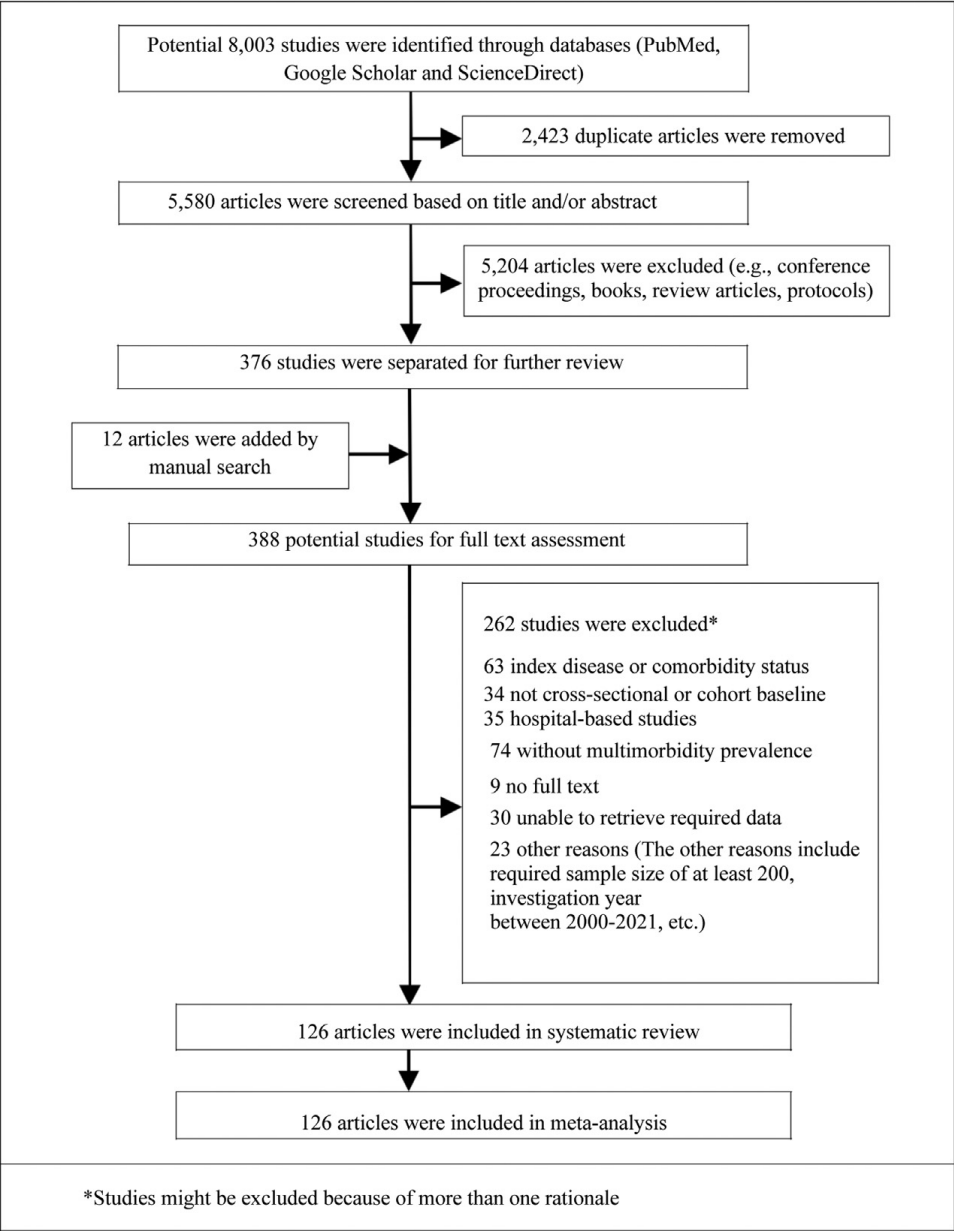
PRISMA flow diagram for study selection.

A flowchart of the literature search to select the relevant articles is summarized in the PRISMA format and is presented in Fig. 1. The initial search retrieved 8003 studies from the three pre-specified databases. After excluding the duplicates, the titles and abstracts were screened for a further selection of probable articles. Subsequently, the investigators selected 376 articles based on eligibility criteria for full-text review. By manual searching through the included papers’ reference lists and reference lists of previous systematic reviews on similar topics, 12 studies were considered for scrutiny, resulting in the total number of potential articles being 388. After excluding 262 studies in full-text review, finally, 126 studies with a total of 15,400,421 (approximately 15.4 million) people were included in the systematic review and meta-analysis. Sample sizes in the studies range from 264 to 3,759,836.^3^^,^^27^^,^^34^, ^35^, ^36^, ^37^, ^38^, ^39^, ^40^, ^41^, ^42^, ^43^, ^44^, ^45^, ^46^, ^47^, ^48^, ^49^, ^50^, ^51^, ^52^, ^53^, ^54^, ^55^, ^56^, ^57^, ^58^, ^59^, ^60^, ^61^, ^62^, ^63^, ^64^, ^65^, ^66^, ^67^, ^68^, ^69^, ^70^, ^71^, ^72^, ^73^, ^74^, ^75^, ^76^, ^77^, ^78^, ^79^, ^80^, ^81^, ^82^, ^83^, ^84^, ^85^, ^86^, ^87^, ^88^, ^89^, ^90^, ^91^, ^92^, ^93^, ^94^, ^95^, ^96^, ^97^, ^98^, ^99^, ^100^, ^101^, ^102^, ^103^, ^104^, ^105^, ^106^, ^107^, ^108^, ^109^, ^110^, ^111^, ^112^, ^113^, ^114^, ^115^, ^116^, ^117^, ^118^, ^119^, ^120^, ^121^, ^122^, ^123^, ^124^, ^125^, ^126^, ^127^, ^128^, ^129^, ^130^, ^131^, ^132^, ^133^, ^134^, ^135^, ^136^, ^137^, ^138^, ^139^, ^140^, ^141^, ^142^, ^143^, ^144^, ^145^, ^146^, ^147^, ^148^, ^149^, ^150^, ^151^, ^152^, ^153^, ^154^, ^155^

### Characteristics of the studies

Table 1 shows the characteristics of the included studies. The 126 population-based studies were conducted across 54 countries. Six of the 126 research included were carried out in multiple countries. The majority of the studies (n = 47) were conducted in Asia, followed by Europe (n = 27), South America (n = 19), Africa (n = 10), North America (n = 14), Oceania (n = 6), and various continents (n = 3). Between 2000 and 2021, 53 studies were carried out in high-income countries (HICs), 48 in upper middle-income countries (UMICs), and 24 in low- and lower-middle-income countries (Low- and LMICs). Most of the studies (121 studies) were cross-sectional in design, and the remaining five had a cohort design, from which we used data from the baseline assessment. When defining multimorbidity, 37 studies looked at 5–9 diseases, 64 studies at 10–19 diseases, and 24 studies at more than 20 diseases.

**Table 1:** Characteristics of the included studies in the meta-analysis (according to the order of year).

Author [Ref]	Country	WB income country	Year of publication	Study period	Study design	Source of data	Ascertainment of morbidities^a^	Sample size	Age, y	Mean/median age, y	Gender (male %)	Number of conditions included	Prevalence, %
Dhungana et al.,^34^	Nepal	Low- or LMIC	2021	2016–2018	Cross-sectional	NCD (non-communicable diseases) survey 2018 in Nepal	Objective	8931	≥20	46.7	42.2	7	14.0
Zhang et al.,^35^	China	UMIC	2021	2017	Cross-sectional	Beijing Longitudinal Study of Aging (BLSA)	Self-reported	1837	≥60	NA	44.3	12	53.2
Keetile et al.,^36^	Botswana	UMIC	2020	2016	Cross-sectional	Survey on Chronic Non-Communicable Diseases in Botswana (NCDs survey)	Self-reported	1178	≥15	NA	30.9	10	5.4
Zou et al.,^37^	China	UMIC	2020	2004–2008	Cross-sectional	A baseline dataset from China Kadoorie Biobank (CKB) study, a Chinese population-based cohort study	Self-reported and Objective	512,888	30–79	NA	41.0	16	15.9
Ma et al.^38^	China	UMIC	2020	2015–2106	Cross-sectional	China Health and Retirement Longitudinal Study (CHARLS)	Self-reported	19,656	≥45	60.2	48.3	14	54.3
Kim et al.,^39^	Korea	HIC	2020	2016	Cross-sectional	Korea National Health and Nutrition Examination Survey (KNHANES)	Self-reported	68,590	≥19	NA	NA	39	23.7
Kshatri et al.^40^	India	Low- or LMIC	2020	2019–2020	Cross-sectional	A cross-sectional study	Self-reported	725	60–106	70.2	52.1	18	48.8
Kyprianidou et al.^41^	Cyprus	HIC	2020	2018–2019	Cross-sectional	A cross-sectional study	Self-reported	1140	≥18	40	43.7	47	28.6
de Melo et al.^42^	Brazil	UMIC	2020	2013–2014	Cross-sectional	National Health Survey database	Self-reported	11,697	≥60	70.1	40.1	13	53.1
Zhang et al.^43^	USA	HIC	2020	2012–2017	Cross-sectional	National Health Interview Survey (2012–2017) of Asian Indians, Chinese, and NHWs (non-Hispanic whites)	Self-reported	132,666	≥18	NA	48.5	10	38.2
Li et al.^44^	China	UMIC	2019	2017	Cross-sectional	A community-based cross-sectional health interview and examination survey	Self-reported and Objective	4833	≥60	NA	45.5	5	16.1
Aminisani et al.^45^	Iran	UMIC	2020	2017–2018	Cross-sectional	Prospective Epidemiological Research Studies in Iran (PERSIAN)	Self-reported	1493	≥50	61.6	38	36	36.6
Craig et al.^46^	Jamaica	Low- or LMIC	2020	2007–2008	Cross-sectional	Jamaica Health and Lifestyle Survey 2007/2008 (JHLS-II)	Self-reported	2551	15–74	NA	NA	11	24.1
Vargese et al.^47^	India	Low- or LMIC	2020	2017	Cross-sectional	A register based cross sectional study	Self-reported	525	≥18	47.4	46.9	12	16.2
Lee et al.^48^	Korea	HIC	2020	2014	Cross-sectional	2014 Korean Health Panel Survey	Self-reported	11,232	≥18	57.5	49.6	≥20	34.8
Zhao et al.^49^	China	UMIC	2020	2011–2015	Cross-sectional	China Health and Retirement Longitudinal Study (CHARLS) for 2011, 2013, and 2015	Self-reported	11,817	≥50	62 (median)	48.8	11	61.9
Wister et al.^50^	Canada	HIC	2020	2010	Cross-sectional	Canadian Longitudinal Study on Aging (CLSA) dataset	Self-reported	15,711	45–85	62	49	27	64
Yao et al.^51^	China	UMIC	2019	2011–2015	Cross-sectional	China Health and Retirement Longitudinal Study (CHARLS)	Self-reported	19,841	≥50	NA	48.6	14	42.4
Zhang et al.^52^	China	UMIC	2019	2015	Cross-sectional	China Health and Retirement Longitudinal Survey (CHARLS) 2015	Self-reported	11,707	≥60	70.5	48.7	14	43.6
Laires et al.^53^	Portugal	HIC	2019	2014	Cross-sectional	Fifth Portuguese National Health Interview Survey, conducted in 2014	Self-reported	15,196	25–79	NA	44	15	43.9
Ba et al.^54^	Vietnam	Low- or LMIC	2019	2018	Cross-sectional	A cross-sectional study	Self-reported	1680	≥15	38	50.1	9	16.4
Khan et al.^55^	Bangladesh	Low- or LMIC	2019	2015–2016	Cross-sectional	A large-scale cross-sectional study	Self-reported	12,338	≥35	58.5	48.6	6	8.4
Singh et al.^56^	South Asia	Low- or LMIC	2018	2010–2011	Cross-sectional	Cardiometabolic Risk Reduction in South Asia Surveillance Study	Self-reported and Objective	16,287	≥20	41	47.3	5	9.4
Lai et al.^57^	Hong Kong	HIC	2019	2008	Cross-sectional	The Thematic Household Survey (THS) on health-related topics	Self-reported	17,396	≥35	NA	48.5	14	8.8
Bao et al.^58^	China	UMIC	2019	NA	Cross-sectional	Cross-sectional community health survey	Self-reported	18,137	≥45	61.4	47.6	19	20.8
Hu et al.^59^	Taiwan	HIC	2019	2003–2013	Cross-sectional	The National Health Insurance Research Database	Self-reported	1,429,527	≥20	NA	NA	20	30.4
Park et al.^60^	Korea	HIC	2019	2013–2015	Cross-sectional	Sixth Korean National Health and Nutrition Examination Survey (KNHANES) conducted in 2013–2015	Self-reported	8370	≥50	62.5	46.3	10	39
Hernandez et al.^61^	Ireland	HIC	2019	NA	Cross-sectional	Irish population study	Self-reported	6101	≥50	NA	46.3	31	73.3
Frolich et al.,^62^	Denmark	HIC	2019	2012	Cross-sectional	Danish national administrative and health registries	Objective	1,397,173	≥16	NA	48.4	16	21.6
Chang et al.,^63^	South Africa	UMIC	2019	2014–2015	Cross-sectional	Population-based survey conducted in The Health and Ageing in Africa: a longitudinal study of an INDEPTH Community in South Africa (HAALSI) Programme	Self-reported and Objective	3889	≥40	61.7	45.2	10	69.4
Nguyen et al.,^64^	England	HIC	2019	2004–2005	Cross-sectional	English Longitudinal Study of Aging (ELSA) wave 2	Self-reported	9171	≥50	66.4	44.5	26	80.8
dos Santos Costa et al.,^65^	Brazil	UMIC	2018	2014	Cross-sectional	Cross-sectional population-based study	Self-reported	1451	≥60	NA	37	29	92.8
Cheung et al.,^66^	Hong Kong	HIC	2018	2016–2017	Cross-sectional	Baseline well-being assessment of the Jockey Club Community eHealth Care Project	Self-reported	2618	≥60	NA	47.5	7	41.8
Zemedikun et al.,^67^	UK	HIC	2018	2006–2010	Cross-sectional	UK Bio-bank, a major collaborative research project	Self-reported and Objective	502,643	40–69	58	45.6	36	19
El Lawindi et al.,^68^	Egypt	Low- or LMIC	2018	2016–2017	Cross-sectional	A community-based cross-sectional study	Self-reported	2317	≥18	36.2	54.9	16	19.6
Stanley et al.,^70^	New Zealand	HIC	2018	2014	Cross-sectional	National-level routine health data on hospital discharges and pharmaceutical dispensing	Objective	3,489,747	≥18	NA	48.2	30	27.9
Araujo et al.,^71^	Brazil	UMIC	2018	2015	Cross-sectional	Cross-sectional population-based study	Self-reported	4001	≥18	NA	47.2	12	29
Jankovic et al.,^72^	Serbia	UMIC	2018	2013	Cross-sectional	2013 National Health Survey (NHS 2013) of the Serbian population	Self-reported	13,765	≥20	51.8	46	13	30.2
Chen et al.,^73^	China	UMIC	2018	2011–2012	Cross-sectional	China Health and Retirement Longitudinal Study 2011	Self-reported	3737	≥45	NA	51.9	16	45.5
Nunes et al.,^74^	Brazil	UMIC	2018	2015–2016	Cross-sectional	The Brazilian Longitudinal Study of Aging (ELSI-Brazil)	Self-reported	9412	≥50	62.9	46	19	67.8
Mondor et al.,^75^	Canada	HIC	2018	2005–2012	Cross-sectional	The Canadian Community Health Survey (CCHS) (2005–2011/12)	Objective	27,195	≥18	NA	48.6	17	33.5
Mounce et al.,^76^	England	HIC	2018	2002–2003	Cohort	The English Longitudinal Study of Aging (ELSA) cohort	Self-reported	4564	≥50	NA	43.7	15	34
Ge et al.,^77^	Singapore	HIC	2018	2015–2016	Cross-sectional	Population Health Index (PHI) survey	Objective	1940	≥21	51.4	43.9	17	35
Camargo-Casas et al.,^78^	Colombia	UMIC	2018	2012	Cross-sectional	Salud, Bienestery, Envejecimiento Bogota (SABE-B), (Health, Well-being and Ageing Study)	Self-reported	2000	≥60	71.1	36.6	12	40.4
Amaral et al.,^79^	Brazil	UMIC	2018	2010	Cross-sectional	A project entitled “Conditions of health, quality of life and depression in elderly persons assisted under the Family Health Strategy in Senador Guiomard, Acre”	Self-reported	264	60–102	NA	39	14	66.3
Puth et al.,^80^	Germany	HIC	2017	2012–2013	Cross-sectional	National telephone health interview survey “German Health Update” (GEDA2012)	Self-reported	19,294	≥18	NA	48.3	17	39.6
Waterhouse et al.,^81^	South Africa	UMIC	2017	2007–2008	Cross-sectional	Wave 1 (2007–08) of the South African Study on Global Ageing and Adult Health	Self-reported and Objective	3055	≥50	NA	39.6	8	12.9
Alimohammadian et al.,^69^	Iran	UMIC	2017	2004–2008	Cross-sectional	Golestan cohort data	Self-reported	49,946	40–75	NA	42.4	8	19.4
Wang et al.,^82^	Australia	HIC	2017	2007	Cross-sectional	2007 National Survey of Mental Health and Wellbeing (NSMHWB)	Self-reported	8820	16–85	44	49.7	8	28.8
Kunna et al.,^83^	China	UMIC	2017	2008–2010	Cross-sectional	World Health Organization Study on Global AGEing and Adult Health (SAGE) Wave 1 (2007–2010)	Self-reported and Objective	11,814	≥50	NA	46.4	8	29.7
Lujic et al.,^84^	Australia	HIC	2017	2005–2009	Cohort	The 45 and Up Study, The PBS (Pharmaceutical Benefits Scheme) database, The NSW (New South Wales) Admitted Patient Data Collection (APDC)	Self-reported	90,352	≥45	70.2	44.3	8	37.4
Nunes et al.,^85^	Brazil	UMIC	2017	2013	Cross-sectional	Population-based data from the Brazilian National Health Survey	Self-reported	60,202	≥18	43.7	44.9	22	22.2
Mini et al.,^86^	India	Low- or LMIC	2017	2011	Cross-sectional	United Nations Population Fund (UNFPA) in the year 2011 on ‘Building Knowledge Base on Population Ageing in India’	Self-reported	9852	≥60	68	47	12	30.7
Larsen et al.,^87^	Denmark	HIC	2017	2013	Cross-sectional	Danish national health survey conducted in 2013	Self-reported	162,283	≥16	47.8	49	15	37
Gu et al.,^88^	China	UMIC	2017	2013	Cross-sectional	A cross-sectional study	Self-reported	2452	≥60	69.2	51.5	13	49.4
Dhalwani et al.,^89^	England	HIC	2017	2008–2013	Cohort	The English Longitudinal Study of Ageing (ELSA) 4, 5, 6	Self-reported	5476	≥50	61 (median)	47	18	21.1
Nunes et al.,^90^	Brazil	UMIC	2016	2012	Cross-sectional	A population-based cross-sectional study	Self-reported	2927	≥20	45.7	41.1	11	29.1
Picco et al.,^91^	Singapore	HIC	2016	2012–2013	Cross-sectional	The Well-being of the Singapore Elderly (WiSE) study	Self-reported	2565	≥60	NA	43.5	10	51.5
Palladino et al.,^92^	16 countries	HIC	2016	2011–2012	Cross-sectional	Survey of Health, Ageing and Retirement in Europe (SHARE) in 2011–12	Self-reported	56,427	≥50	66	44.1	13	37.3
Cossec et al.,^93^	France	HIC	2016	2012	Cross-sectional	Health, Health Care and Insurance Survey from 2012 (Enquête Santé et Protection Sociale) called ESPS	Self-reported	4236	56–105	69.6	43	7	14.9
Vadrevu et al.,^104^	India	Low- or LMIC	2016	2009	Cross-sectional	A cross-sectional survey	Self-reported	815	≥40	54.9	51.3	6	44.1
Marengoni et al.,^95^	Sweden	HIC	2016	2001–2004	Cross-sectional	Swedish National study on Aging and Care in Kungsholmen (SNAC-K)	Objective	3155	≥60	74.4	35.7	≥5	52.4
Jovic et al.,^96^	Serbia	UMIC	2016	2013	Cross-sectional	2013 National Health Survey (NHS 2013) of the Serbian population	Self-reported	13,103	≥20	49.4	48.1	12	26.9
Su et al.,^97^	China	UMIC	2016	2013	Cross-sectional	A large-scale survey initiated by Shanghai Health and Family Planning Commission	Self-reported	2058	≥80	NA	42.1	10	49.2
Ramond-Roquin et al.,^98^	Canada	HIC	2016	2010	Cross-sectional	The Program of Research on the Evolution of a Cohort Investigating Health System Effects (PRECISE)	Self-reported	1710	25–75	51.3	40.5	21	63.8
Lenzi et al.,^99^	Italy	HIC	2016	2012	Cross-sectional	The hospital discharge record (HDR) database, the mental health information system, residential mental healthcare discharge records, the outpatient pharmaceutical database, the regional mortality register database	Objective	3,759,836	≥18	NA	48	26	15.3
Dung et al.,^100^	Vietnam	Low- or LMIC	2016	2011	Cross-sectional	Vietnam Ageing Survey (VNAS)	Self-reported	2789	≥60	71.9	39.7	12	43.9
Valadares et al.,^101^	Brazil	UMIC	2016	2012–2013	Cross-sectional	Cross-sectional population-based study	Self-reported	749	45–60	52.5	0	11	53
Pache et al.,^102^	Switzerland	HIC	2015	2003–2006	Cross-sectional	Population-based study	Objective	3714	35–75	49.6	47	27	56.3
Afshar et al.,^103^	28 countries	NA	2015	2003	Cross-sectional	World Health Survey (2003)	Self-reported	125,404	≥18	NA	48.5	6	7.8
Roberts et al.,^104^	Canada	HIC	2015	2011–2012	Cross-sectional	Canadian Community Health Survey 2011/12	Self-reported	105,406	≥20	NA	44.1	9	12.9
Arokiasamy et al.,^105^	6 Countries	Low- or LMIC	2015	2007–2010	Cross-sectional	World Health Organization Study on Global AGEing and Adult Health (SAGE) Wave 1 (2007–2010)	Self-reported and Objective	42,236	≥18	NA	50.7	8	21.9
Ha et al.,^106^	Vietnam	Low- or LMIC	2015	2010	Cross-sectional	Population-based study	Objective	2400	≥60	72.6	34.8	6	39.2
Wang et al.,^107^	China	UMIC	2015	2012	Cross-sectional	Jilin Provincial Chronic Disease Survey	Self-reported	21,435	18–79	NA	NA	18	24.7
Wang et al.,^108^	China	UMIC	2015	2010–2011	Cross-sectional	Confucius Hometown Aging Project in Shandong, China (June 2010–July 2011)	Self-reported and Objective	1480	≥60	68.5	40.6	16	90.5
Nunes et al.,^109^	Brazil	UMIC	2015	2008	Cross-sectional	A population-based cross-sectional study	Self-reported	1593	≥60	NA	37.2	17	81.3
Chung et al.,^110^	Hong Kong	HIC	2015	2011–2012	Cross-sectional	Thematic Household Survey (THS) conducted by the Census and Statistics Department (C&SD) of the Hong Kong SAR Government	Self-reported	25,780	≥15	NA	47.8	46	12.5
Hussain et al.,^3^	Indonesia	UMIC	2015	2007–2008	Cross-sectional	Fourth wave of Indonesian Family Life Survey (IFLS-4)	Self-reported and Objective	9438	≥40	NA	48.4	11	35.7
Ruel et al.,^111^	Australia	HIC	2014	2000–2002	Case-sectional	North West Adelaide longitudinal Health Study (NWAHS)	Self-reported and Objective	1854	≥18	50	48	8	32
Mahwati et al.,^112^	Indonesia	UMIC	2014	2007–2008	Cross-sectional	The fourth survey of the Indonesian Family Life Survey (IFLS) which held in 2007	Self-reported	2960	≥60	NA	46	9	15.8
Islam et al.,^27^	Australia	HIC	2014	2009	Cross-sectional	A cross-sectional survey	Self-reported	4574	≥50	69.3	NA	11	52
Banjare et al.,^113^	India	Low- or LMIC	2014	2011–2012	Cross-sectional	A cross-sectional survey	Self-reported	310	≥60	NA	49.4	21	56.8
Hien et al.,^114^	Burkina Faso	Low- or LMIC	2014	2012	Cross-sectional	Cross-sectional study among community-dwelling elderly	Objective	389	≥60	69	55.3	15	65
Orueta et al.,^115^	Spain	HIC	2013	2007–2011	Cross-sectional	Primary care electronic medical records, hospital admissions, and outpatient care databases	Objective	452,698	≥65	NA	42.5	47	61.1
Aguiar et al.,^116^	Brazil	UMIC	2013	2011	Cross-sectional	A cross-sectional, population-based study	Self-reported	622	≥50	64.1	0	12	58.2
Alaba et al.^117^	South Africa	UMIC	2013	2008	Cross-sectional	South African National Income Dynamics Survey (SA-NIDS) of 2008	Self-reported	11,638	≥18	40	39	6	4
Wu et al.,^118^	China	UMIC	2013	2010	Cross-sectional	SAGE-China Wave 1	Self-reported and Objective	13,157	≥50	62.6	48.1	8	18.9
Phaswana-Mafuya et al.,^119^	South Africa	UMIC	2013	2008	Cross-sectional	National population-based cross-sectional survey	Self-reported	3638	≥50	NA	42.5	8	22.5
Jerliu et al.,^120^	Kosovo	UMIC	2013	2011	Cross-sectional	A nationwide cross-sectional study	Self-reported	1890	≥65	73.4	50.2	6	45.2
Kiliari et al.,^121^	Cyprus	HIC	2013	2008	Cross-sectional	A nationally based survey	Self-reported	465	18–88	53	43.2	27	28.5
Fuchs et al.,^122^	Germany	HIC	2012	2008–2009	Cross-sectional	Telephone health interview surveys in representative samples of the German adult population (German Health Update, GEDA)	Self-reported	21,262	18–100	48.8	48.5	22	40.1
MacHado et al.,^123^	Brazil	UMIC	2012	2005	Cross-sectional	A secondary analysis of a cross-sectional population-based study	Self-reported	377	40–65	NA	0	5	39.3
Kirchberger et al.,^124^	Germany	HIC	2012	2008–2009	Cross-sectional	The population-based KORA-Age project	Self-reported	4067	65–94	73.4	48.8	13	58.6
Agborsangaya et al.,^125^	Canada	HIC	2012	2010	Cross-sectional	Health Quality Council of Alberta (HQCA) 2010 Patient Experience Survey	Self-reported	5010	≥18	46.7	47.7	16	19
Tucker-Seeley et al.,^126^	USA	HIC	2011	2004	Cross-sectional	The Health and Retirement Study (HRS)	Self-reported	7305	≥50	65	46.4	6	35.4
Khanam et al.,^127^	Bangladesh	Low- or LMIC	2011	2004	Cross-sectional	A descriptive cross-sectional study	Objective	452	60–92	69.5	45.1	9	53.8
Taylor et al.,^128^	Australia	HIC	2010	2004–2006	Cross-sectional	North West Adelaide Health Study (NWAHS Stage 2)	Self-reported and Objective	3206	≥20	NA	NA	7	17.1
Loza et al.,^129^	Spain	HIC	2009	1999–2000	Cross-sectional	A national health survey	Self-reported and Objective	2192	≥20	NA	46.3	9	29.7
Minh et al.,^130^	5 countries	Low- or LMIC	2008	2005	Cross-sectional	2005 cross-site study of 8 sites in 5 Asian countries	Self-reported	18,494	25–64	NA	50	7	7.2
Camargo-Casas,^78^	Columbia	UMIC	2018	2012	Cross-sectional	NA	Self-reported	2000	≥60	71.1	36.6	NA	40.4
Wilk et al.^131^	Canada	HIC	2021	2015–2018	Cross-sectional	Canadian Community Health Survey (CCHS), 2015–2018	Self-reported	100,803	≥20	47.9	48.9	5	8.1
Tomita et al.^132^	Tanzania	Low- or LMIC	2021	2017–2018	Cross-sectional	The Dar es Salaam Health and Demographic Surveillance System (HDSS)	Self-reported	2299	≥40	53.0	32.4	8	24.8
Smith et al.^133^	Ireland	HIC	2021	2009–2013	Cross-sectional	Irish Longitudinal Study on Ageing (TILDA) Survey	Self-reported	5946	≥50	62.7	51.7	14	50.3
Delpino et al.,^134^	Brazil	UMIC	2021	2019	Cross-sectional	The Brazilian National Health Survey 2019	Self-reported	65,803	18–59	NA	47.8	14	22.3
Marthias et al.,^135^	Indonesia	UMIC	2021	2014	Cross-sectional	The Indonesian Family Life Survey 2014 (Wave – 5)	Self-reported and Objective	3678	≥50	65 (median)	46.1	10	22.0
Zhang et al.^136^	China	UMIC	2021	2019	Cross-sectional	A cross-sectional study	Self-reported and Objective	3250	≥60	NA	46.6	26	30.3
Lin et al.,^137^	Taiwan	HIC	2021	2017–2019	Cross-sectional	A community-based survey	Self-reported	3739	65–85	72.9	42.8	7	27.8
Nicholson et al.,^138^	Canada	HIC	2021	2015	Cross-sectional	The Canadian Longitudinal Study on Aging (CLSA)	Self-reported	11,161	65–85	NA	47.5	15	75.3
Bezerra et al.,^139^	17 countries	HIC	2021	2015	Cross-sectional	Survey of Health, Aging and Retirement in Europe (SHARE) 2015 (Wave – 6)	Self-reported	63,844	≥50	NA	44.3	13	33.6
Koyanagi et al.,^140^	48 countries	Low- or LMIC	2021	2002–2004	Cross-sectional	The World Health Survey 2002–2004	Self-reported	224,842	≥18	38.3	49.3	10	3.8
Shi et al.,^141^	Brazil	UMIC	2021	1998–2013	Cross-sectional	The National Sample Household and Brazilian National Health Survey	Self-reported	795,271	≥18	NA	47.2	9	18.3
Wang et al.,^142^	China	UMIC	2021	2018	Cross-sectional	A cross-sectional survey	Self-reported	1871	≥60	83.6	39.0	33	74.3
He et al.,^143^	China	UMIC	2021	2014–2019	Cohort	Annual health examination data set in the Xinzheng electronic health Management	Self-reported and Objective	50,100	≥65	69.2 (median)	46.1	7	31.4
Ballesteros et al.,^144^	Colombia	UMIC	2021	2015	Cross-sectional	Colombian population-based survey Health, Wellbeing and Aging (Salud, Bienestar y Envejecimiento—SABE)	Self-reported	17,571	≥60	69.2	44.3	10	62.3
Mohamed et al.,^145^	Kenya	LMIC	2021	2003–2015	Cross-sectional	Nairobi Urban Health & Demographic Surveillance System (NUHDSS)	Self-reported and Objective	2003	≥40	48.8	46.0	16	28.7
Kanungo et al.,^146^	India	Low- or LMIC	2021	2017–2019	Cross-sectional	Longitudinal Ageing Study in India (LASI), Wave-1	Self-reported	59,764	45–116	60.2	45.9	12	50.4
Oh et al.,^147^	USA	HIC	2020	2001–2003	Cross-sectional	The National Survey of American Life	Self-reported	5191	≥18	42.2	63.1	22	54.1
King et al.,^148^	USA	HIC	2019	2013–2014	Cross-sectional	The National Health and Nutrition Examination Survey (NHANES)	Self-reported and Objective	5541	≥20	NA	48.2	11	59.6
Bowling et al.^149^	USA	HIC	2019	2011–2016	Cross-sectional	The National Health and Nutrition Examination Survey (NHANES), 2011–2016	Self-reported and Objective	4217	≥50	56.7	48.7	12	72.4
Keats et al.^150^	Canada	HIC	2017	2009–2015	Cohort	Atlantic Partnership for Tomorrow's Health (PATH) study	Self-reported	18,709	≥35	NA	30.0	18	38.2
Quinaz Romana et al.^151^	Portugal	HIC	2019	2013–2016	Cross-sectional	The National Health Examination Survey (INSEF)	Objective	4911	≥25	NA	47.5	20	38.3
de Souza et al.^152^	Brazil	UMIC	2019	2001–2002	Cohort	A longitudinal study of municipal technical and administrative employees in Rio de Janeiro	Self-reported and Objective	733	≥24	41.6	33.8	15	45.6
Costa et al.^153^	Brazil	UMIC	2020	2013–2014	Cross-sectional	Brazilian National Survey	Self-reported and Objective	23,329	≥20	37.9	47.2	14	10.9
Keomma et al.^154^	Brazil	UMIC	2020	2015	Cross-sectional	The ISA-Capital health survey	Self-reported and Objective	1019	≥60	67.7	40.3	10	40
Jürisson et al.^155^	Estonia	HIC	2021	2015–2017	Cross-sectional	Estonian Health Insurance Fund	Objective	909,477	≥25	53.4	45.9	55	39.8

a Ascertainment of morbidities- Objective: medical records/clinical examinations.

### Global and regional prevalence of multimorbidity

**Fig. 2:**
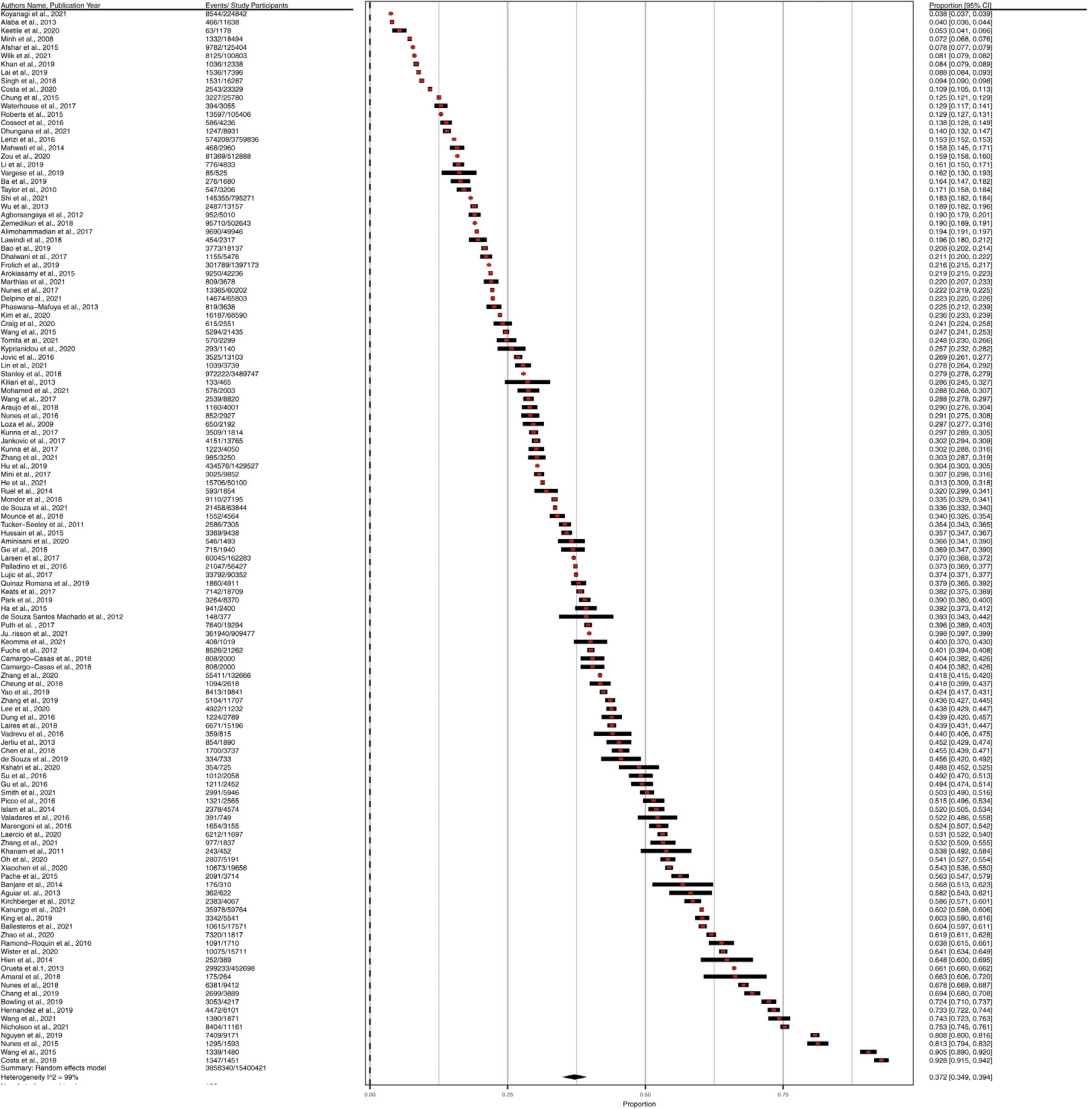
Forest Plot of the Overall Prevalence of multimorbidity in community settings.

The prevalence of multimorbidity among the adult population ranged from 4.0% to 92.8% in the studies. Prevalence estimates along with confidence intervals for multimorbidity are shown in Fig. 2 by using a forest plot. The random-effects overall pooled estimated (126 studies) prevalence of multimorbidity was 37.2% (95% CI = 34.9%–39.4%, I^2^ = 99.7%). The pooled proportion of multimorbidity was the highest in South America with 45.7% (95% CI = 39.0%–52.5%, I^2^ = 99.0%). On the other hand, the pooled prevalence of multimorbidity was the lowest in Africa with 28.2% (95% CI = 15.6%–40.8%, I^2^ = 99.0%). However, studies from Asia, Europe, North America, and Oceania were calculated to have the pooled prevalence of multimorbidity 35% (95% CI = 31.4%–38.5%, I^2^ = 99.3%), 39.2% (95% CI = 33.2%–45.2%), 43.1% (95% CI = 32.3%–53.8%), and 32.5% (95% CI = 26.8%–38.2%, I^2^ = 98.9%), respectively.

### Subgroup analysis

**Fig. 3:**
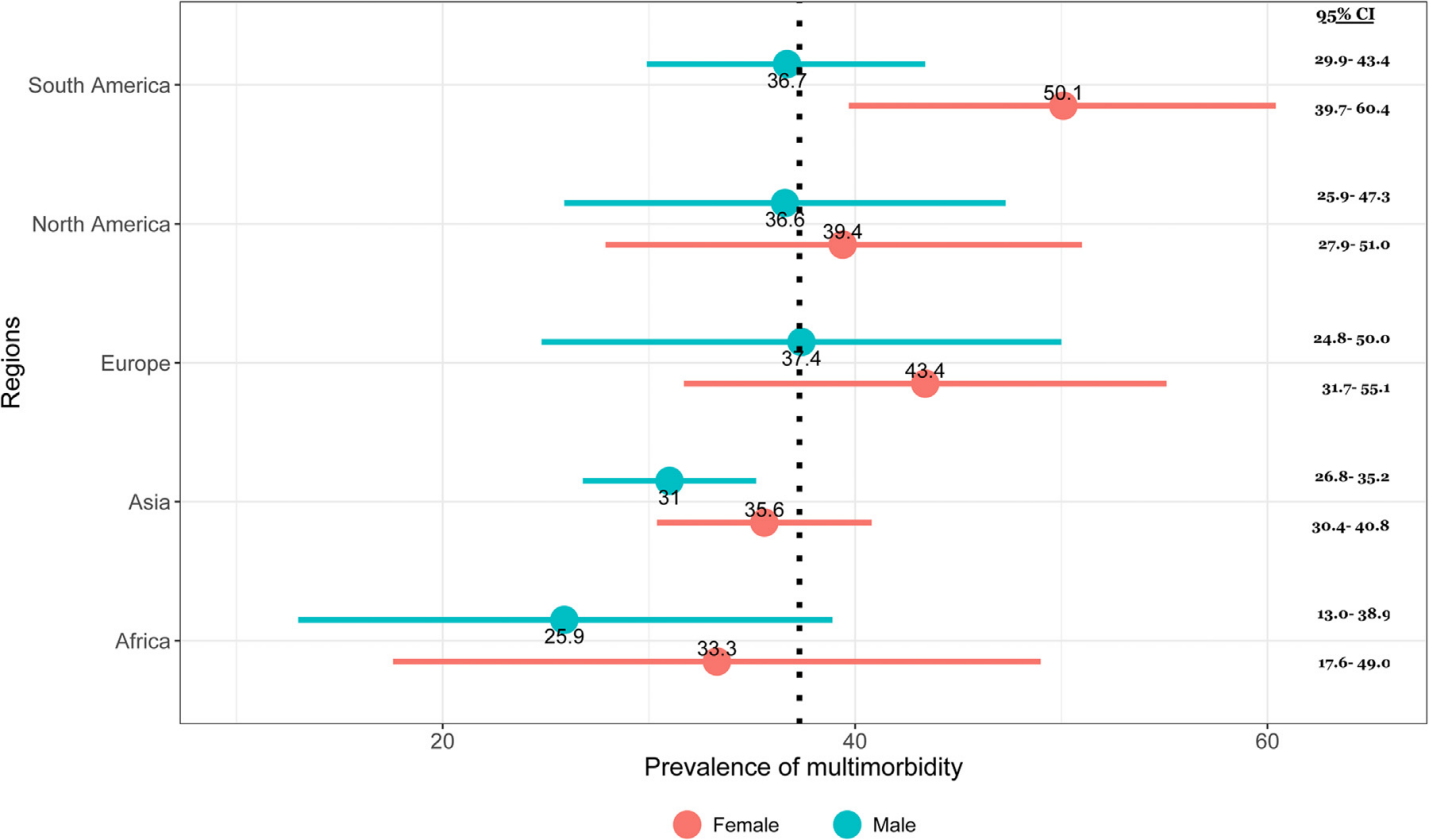
Regional differences of pooled prevalence of multimorbidity by gender.

The subgroup analysis of the prevalence of multimorbidity by continents, study design, number of diseases included in multimorbidity, age, and gender is shown in Table 2. The forest plots are given in the Supplementary File 3. Of note, 85 studies reported the prevalence of multimorbidity in males and females. According to the table, the pooled prevalence of multimorbidity was higher among female participants (39.4%, 95% CI = 36.4–42.4%, I^2^ = 99.6%) than male participants (32.8%, 95% CI = 30.0–35.6%, I^2^ = 99.6%). The Fig. 3 shows the gender segregation of pooled prevalence of multimorbidity by geographic regions. Female participants from South America (prevalence 50.1% and 95% CI = 39.7–60.4%) appeared to have the most multimorbid conditions in the world. Multimorbid illnesses were notably more prevalent in European and North American women than in male participants.

**Table 2:** Summary results of subgroup analysis.

Subgroup		No of studies	Weighted Mean age^a^ (SE)	Pooled prevalence of Multimorbidity	95% CI	I^2^ (%)
WHO geographic Region	Africa	10	49.71 (10.9)	0.282	0.156–0.408	99.9
	Asia	47	57.76 (11.6)	0.350	0.314–0.385	99.9
	Europe	27	58.16 (9.6)	0.392	0.332–0.452	99.6
	North America	14	54.61 (6.1)	0.431	0.323–0.538	99.9
	Oceania	6	58.38 (13.3)	0.325	0.268–0.382	98.3
	South America	19	56.38 (13.4)	0.457	0.390–0.525	99.9
WB/WHO income region	High	53	56.61 (9.7)	0.386	0.353–0.419	99.9
	Upper-middle	48	60.43 (12.5)	0.387	0.355–0.419	99.9
	Low and Low-middle	24	53.19 (11.93)	0.321	0.243–0.40	99.8
Study design	Cross-sectional	121	56.46 (11.06)	0.374	0.351–0.396	99.3
	Cohort	5	62.7 (6.71)	0.324	0.279–0.369	96.7
Number of conditions included for defining multimorbidity	5–9 conditions	37	57.54 (12.64)	0.250	0.223–0.278	97.9
	10–19 conditions	64	60.15 (9.96)	0.413	0.376–0.450	99.9
	≥20 conditions	24	53.44 (8.47)	0.457	0.393–0.500	99.9
Gender	Female	85	–	0.394	0.364–0.424	99.9
	Male	85	–	0.328	0.300–0.356	99.2
Mental health included in Multimorbidity definition	Yes	91	57.62 (11.02)	0.384	0.359–0.410	99.3
	No^b^	28	61.12 (11.56)	0.332	0.271–0.392	98.9
Age of the study participants	≥30 years	76	65.2 (6.26)	0.444	0.393–0.494	99.9
	≥40 years	71	65.86 (5.69)	0.457	0.402–0.512	99.9
	≥50 years	58	67.42 (4.63)	0.472	0.420–0.525	99.9
	≥60 years	33	70.91 (2.01)	0.510	0.441–0.580	98.3
Overall		126	56.95 (10.85)	0.373	0.349–0.394	99.0

a The weighted mean age and standard error (SE) were calculated based on the available study sample size and the study participant's mean/median age.

b Because the disease list was not mentioned in a few of the articles, we assumed these articles may not contain mental health.

Based on the continents of the studies, the estimated pooled prevalence of multimorbidity was found 38.6% (95% CI = 35.3%–41.9%, I^2^ = 99.2%) in high-income countries, 38.7% (95% CI = 35.5–41.9%, I^2^ = 99.2%) in upper middle-income countries (UMICs), and 32.1% (95% CI = 24.3–40.0%, I^2^ = 99.5%) in Low- and LMICs. In the case of the number of diseases included in the multimorbidity, the prevalence was found 44.7% (95% CI = 39.5%–50.0%, I^2^ = 99.3%) among the studies that considered ≥20 diseases. The prevalence of multimorbidity was 25.0% (95% CI = 22.3–27.8%, I^2^ = 99.0%) for studies with 5–9 diseases, and 41.3% (95% CI = 37.6%–45.0%, I^2^ = 99.0%) for studies with 10–19 diseases. When mental health is included in the multimorbidity definition, the prevalence (38.4%, 95% CI = 35.9–41.0%, I^2^ = 99.0%) was higher than without inclusion of mental health (33.2%, 95% CI = 27.1–39.2%, I^2^ = 99.1%).

Among the different age groups of the study participants, the highest prevalence was found in the studies that included the respondents more than 60 years with 51.0% (95% CI = 44.1%–58.0%). The pooled prevalence was 44.4% (95% CI = 39.3%–49.4%, I^2^ = 99.1%) among the participants with 30 years and above. When the study participants were ≥40 years and ≥50 years, the pooled proportion of multimorbidity was 45.7% (95% CI = 40.2%–51.2%, I^2^ = 99.0%) and 47.2% (95% CI = 42.0%–52.5%, I^2^ = 99.1%), respectively.

There was a difference in the prevalence of multimorbidity by study design among the studies. The pooled prevalence of multimorbidity was 37.4% (95% CI = 35.1%–39.6%, I^2^ = 99.0%) for cross-sectional studies, and 32.4% (95% CI = 27.9%–36.9%, I^2^ = 96.7%) for cohort studies.

### Trends of global multimorbidity prevalence over time

**Fig. 4:**
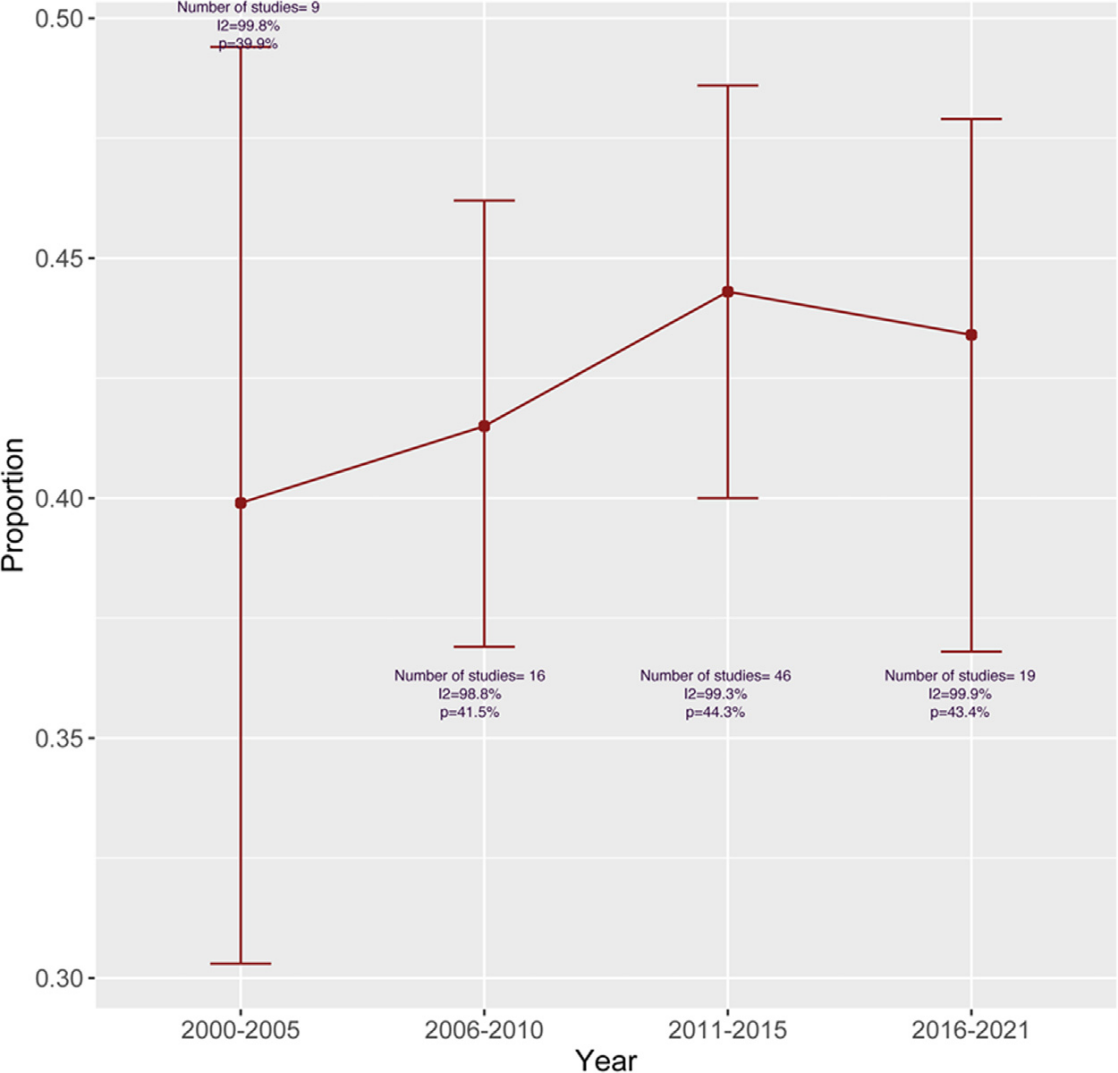
Pooled prevalence of multimorbidity by year.

The global prevalence of multimorbidity by 5-year interval is displayed in Fig. 4, considering studies that contains 10 or more diseases. The five-year span was categorized based on the year in which investigations were done. If a study was completed between 2013 and 2016, we assumed it was conducted between 2011 and 2015 because the majority of years fell within the interval. The study was removed from the analysis if it did not belong to any of the groups. We excluded papers that reported a multimorbidity prevalence of less than 10% or greater than 80% in order to minimize variability in trend analysis. The prevalence of multimorbidity has been on the rise globally since 2000, but it has remained rather stable since 2011. The trend analysis with the studies that considered ten or more illnesses in multimorbidity classifications, showed that the global prevalence of multimorbidity remained high, exceeding 40%.

### Sensitivity analysis for global prevalence

We conducted sensitivity analyses including studies with more than 1000 participants, removing studies from Africa, and removing studies that showed prevalence of less than 20% and more than 80%. The reasons for removing studies with less than 1000 participants are to increase estimate reliability and precision of the estimate with the studies with a larger sample size. Furthermore, we excluded papers with extreme prevalence estimates of less than 20% and more than 80% because these values could lead to heterogeneity in predicting worldwide prevalence. Forest plots are reported in Supplementary File 4. When considering studies of more than 1000 participants, the global prevalence among participants tends to be 36.1% (95% CI = 33.7–38.4%, I^2^ = 98.8%), which is in line with the findings of the meta-analysis with 126 studies. After excluding African studies, the prevalence was 37.9% (95% CI = 35.4%–40.2%), which is comparable to the meta-analysis with 126 studies. We also found the global prevalence was higher than the overall pooled prevalence after removing studies with extreme prevalence. The results showed the prevalence 42.3% (95% CI = 39.8–44.7%, I^2^ = 98.8%) after excluding studies with extreme prevalence. The findings excluding studies with extreme prevalence are, therefore, higher than the meta-analysis of 126 studies. With high-quality papers (minimal bias according to NOS), we found the prevalence to be 36.6% (95% CI = 33.6–39.5%, I^2^ = 99.8), which imply a similar result that we analyzed in the meta-analysis of 126 studies. Moreover, the studies using self-reported multimorbid data indicate a prevalence of 38.3% (95% CI = 35.1–41.5%), but the studies with data from medical records indicate a prevalence of 34.3% [95% CI = 30.3–38.2%].

### Publication bias

The Egger test found that there was no statistically significant publication bias (P > .05) among the 83 population-based studies evaluating the relationship between gender and multimorbidity status. However, the Egger test revealed a statistically significant publication bias among the 126 population-based studies for proportion (Supplementary File 5). We also have applied trim-and-fill method to adjust for this publication bias in the analysis. We see that the procedure identified and trimmed 42 added studies. The overall effect estimated by the trim-and-fill is 26.71% (95% CI = 0.2350–0.2799). Our initial estimate with 126 studies was 37.1%, which is substantially larger than the bias-corrected effect. If we assume that publication bias affected our findings, the trim-and-fill method allows us to hypothesize that our initial results were overstated because of publication bias, and the global estimate when controlling for selective publication might be 26.71%. Moreover, considering the odds ratio in a funnel plot we found a high existence of publication bias in our study. Consequently, publication bias may be a cause of heterogeneity in investigating overall proportion.

## Discussion

This study analyzed data from 126 studies that involved nearly 15.4 million people from 54 countries, providing an up-to-date global multimorbidity prevalence of 37.2% (95% CI = 34.9–39.4%). A previous meta-analysis with studies until 2017 found that 33.1% had multimorbidity in the adult population aged 18 and older living in the community.^12^ In comparison to that meta-analysis including studies in community settings, we found a higher prevalence of multimorbidity. Another meta-analysis that included studies from both community and healthcare settings estimated the overall prevalence of multimorbidity was 42.4% (95% CI = 38.9–46.0%) among adults.^156^ The inclusion of studies from primary care and health care settings in the meta-analysis resulted in a higher pooled prevalence than ours.

The sub-group analysis by region showed significant differences in the pooled prevalence of multimorbidity. Our analysis showed that the prevalence of multimorbidity was highest in South America. The result is consistent with a meta-analysis that found that the pooled proportion of multimorbidity in Latin America and the Caribbean was as high as 43% (95% CI: 35–51%).^157^ Africa had the lowest prevalence of multimorbidity, according to our analysis. The result could be attributable to the low age group of participants in the African studies compared to other geographic regions. The lowest rate of multimorbidity in Africa should be interpreted with caution because it raises the possibility that many people living with chronic illnesses in Africa are going undiagnosed.

In subgroup analysis, the prevalence of multimorbidity was lower in Low- and LMICs than in UMICs and HICs. The prevalence of multimorbidity was highest in UMICs. This difference is consistent with another study's findings, where a meta-analysis in community settings found that the pooled multimorbidity prevalence was higher in HICs than LMICs.^12^^,^^156^ The majority of the survey included in the meta-analysis were from HICs and UMICs, with a few studies conducted in Low-income countries. It may reflect the differences in diagnostic and data management systems among HICs, UMICs, and Low- and LMICs. According to a study, the disparity in prevalence estimates between HICs and LMICs could be due to the fewer publications on multimorbidity prevalence in LMICs because of limited understanding and importance of multimorbidity in LMICs compared to HICs.^158^ People in low-income countries may be less likely to seek treatment for diseases than those in high-income countries. Therefore, the prevalence in low-income countries may be underestimated if diseases are defined using medical records.

The pooled prevalence of multimorbidity was higher for the cross-sectional study design than for the cohort study type in this meta-analysis. This disparity in multimorbidity prevalence could be due to study designs with varying levels of methodological differences, such as various study populations, sampling procedures, sample coverage, sample sizes, data collection, and so on. Besides, we considered the baseline sample for a cohort study design that might contribute to the lower prevalence.

For included studies, the more the number of diseases evaluated for multimorbidity, the higher the prevalence. When examining 20 or more conditions for multimorbidity, the prevalence was 44.7%, but it was lowered to 41.3% for 10–19 diseases and 25.0% for 5–9 diseases to define multimorbidity. According to a study, the different combinations of illnesses may cause the prevalence of multimorbidity to differ significantly.^156^^,^^159^ A range of different combinations of multimorbidity definitions has been proposed in the literature, ranging from a list of 16 chronic diseases to 291 diseases.^156^^,^^158^, ^159^, ^160^, ^161^ Furthermore, the pooled estimate of multimorbidity prevalence with the studies those included mental health in the definition of multimorbidity was greater. Previous studies identified a correlation between multimorbidity and mental health.^20^^,^^162^^,^^163^ Our findings, the higher prevalence of multimorbidity with the studies that included mental health, reveal consistency with the findings of previous research.

Our study showed that prevalence estimates varied substantially according to age and gender. Our research showed that females had a higher pooled prevalence of multimorbidity than males. It indicates an association between gender and multimorbidity (evidence of which was provided in multiple studies).^69^^,^^162^^,^^163^ According to our findings, multimorbidity increases with age. While the prevalence estimates varied between and within age groups, our meta-analysis indicated that a large proportion of individuals over 60 had multimorbidity. It is well established that the prevalence of multimorbidity increases in very old persons.^164^, ^165^

The calculation of the global prevalence of multimorbidity based on the study's publication interval of 5-year is one of the most important findings of our research. According to our findings, the prevalence of multimorbidity has changed considerably over the previous two decades but has remained relatively consistent since 2011. This suggests a gradual decline in the global burden of multimorbidity. The plateau observed in multimorbidity prevalence since 2011 may be attributable to a handful of the 19 studies that showed low prevalence in 2016–2021. Therefore, this conclusion should be studied further. Over the years, the global prevalence of multimorbidity among adults has exceeded 40 percent, indicating a high burden of multimorbidity exists over years.

One of the study's strengths was its strong study selection and screening protocols. Because of our rigorous search approach and inclusion criteria, we were able to conduct the largest systematic review of multimorbidity prevalence in community settings to date. The majority of the papers included in the review were of high quality. The comprehensive subgroup analyses demonstrate that our findings are applicable to a wide range of contexts. One important finding of our study is the estimation of the global prevalence of multimorbidity by year of publication. This review did, however, have several limitations. To report multimorbidity prevalence, the majority of the studies in our sample used self-reported data. As a result, such research findings were prone to response bias. High heterogeneity between studies in our meta-analysis implies that the prevalence of multimorbidity varies between studies. To overcome this constraint, we used a random-effects model and performed subgroup analyses. Furthermore, considerable heterogeneity may indicate that the prevalence of multimorbidity varies significantly by geographical region, country income classification, gender, age group, number of diseases considered for multimorbidity, or study methodology.

The high prevalence of multimorbidity highlights the need for healthcare reforms and improvements in several continents. Policymakers should commit to increasing multimorbidity awareness, particularly in relation to mental health management, supporting innovation, maximizing the use of existing resources, and coordinating the efforts of multiple countries to reduce the burden and fatal effects of multimorbidity. More than half of the global adult population over the age of 60 has multimorbid illnesses, and female adults are more prone to develop multimorbidity than male adults. Therefore, management should incorporate these findings into healthcare policies, and countries, particularly in South America, should aim to increase their preventative efforts and build more integrated care models to reduce the burden.

## Contributors

A.H., S.R.C., D.C.D., and T.C.S. contributed to the study concept, literature search, and design. A.H., S.R.C., D.C.D., T.C.S. and J.B. contributed to the data acquisition. A.H. and S.R.C. accessed the data and contributed to the data analysis. A.H., S.R.C., and J.B. contributed to the data interpretation. A.H., S.R.C. and D.C.D. drafted the manuscript. All authors contributed to the critical revision of the manuscript.

## Data sharing statement

Because this meta-analysis was based on data extracted from previously published research, most of the data and study materials are available in the public domain. For further discussions, we invite interested parties to contact the corresponding author.

## Declaration of interests

All other authors declare no competing interests.

## Figures and Tables

**Appendix A:** Search strategy.

A. PubMed		
#1	(“Prevalence” OR “Surveillance” OR “Surveys” OR “Epidemiology”) AND (“Multimorbidity” OR “Multi-morbidity” OR “Multimorbidities” OR “Multi-morbidities” OR “Multi morbidity” OR “Multi morbidities”)	3734
#2	(“Risk factors” OR “Determinants” OR “Predictors”) AND (“Multimorbidity” OR “Multi-morbidity” OR “Multimorbidities” OR “Multi-morbidities” OR “Multi morbidity” OR “Multi morbidities”)	1500
#3	(“Aging” OR “Gender”) AND (“Multimorbidity” OR “Multi-morbidity” OR “Multimorbidities” OR “Multi-morbidities” OR “Multi morbidity” OR “Multi morbidities”)	1708
Searching date starting from 2000/01/01 to 2021/12/31All the entries were under ‘All fields’ categories		
B. Google Scholar		
#1	(“Prevalence” OR “Surveillance” OR “Surveys” OR “Epidemiology”) AND (“Multimorbidity” OR “Multi-morbidity” OR “Multimorbidities” OR “Multi-morbidities” OR “Multi morbidity” OR “Multi morbidities”)	18,913
#2	(“Risk factors” OR “Determinants” OR “Predictors”) AND (“Multimorbidity” OR “Multi-morbidity” OR “Multimorbidities” OR “Multi-morbidities” OR “Multi morbidity” OR “Multi morbidities”)	16,500
#3	(“Aging” OR “Gender”) AND (“Multimorbidity” OR “Multi-morbidity” OR “Multimorbidities” OR “Multi-morbidities” OR “Multi morbidity” OR “Multi morbidities”)	17,103
Searching date starting from 2000/01/01 to 2021/12/31		
C. ScienceDirect		
#1	(“Prevalence” OR “Surveys” OR “Epidemiology”) AND (“Multimorbidity” OR “Multi-morbidity” OR “Multimorbidities” OR “Multi-morbidities” OR “Multi morbidity” OR “Multi morbidities”)	4104
#2	(“Risk factors” OR “Determinants” OR “Predictors”) AND (“Multimorbidity” OR “Multi-morbidity” OR “Multimorbidities” OR “Multi-morbidities” OR “Multi morbidity” OR “Multi morbidities”)	4133
#3	(“Aging” OR “Gender”) AND (“Multimorbidity” OR “Multi-morbidity” OR “Multimorbidities” OR “Multi-morbidities” OR “Multi morbidity” OR “Multi morbidities”)	4391
Searching date starting from 2000/01/01 to 2021/12/31		
D. Embase		
#1	(“Prevalence” OR “Surveillance” OR “Surveys” OR “Epidemiology”) AND (“Multimorbidity” OR “Multi-morbidity” OR “Multimorbidities” OR “Multi-morbidities” OR “Multi morbidity” OR “Multi morbidities”)	8713
#2	(“Risk factors” OR “Determinants” OR “Predictors”) AND (“Multimorbidity” OR “Multi-morbidity” OR “Multimorbidities” OR “Multi-morbidities” OR “Multi morbidity” OR “Multi morbidities”)	3616
#3	(“Aging” OR “Gender”) AND (“Multimorbidity” OR “Multi-morbidity” OR “Multimorbidities” OR “Multi-morbidities” OR “Multi morbidity” OR “Multi morbidities”)	7138
Searching date starting from 2000/01/01 to 2021/12/31		

## Appendix A Supplementary data

Supplementary File 1 S1. Study quality assessment details for cohort, and cross-sectional studies by New-Castle Ottawa Scale.

Supplementary File 2 S2. Preferred Reporting Items for Systematic Reviews and Meta-analysis (PRISMA) checklist.

Supplementary File 3 Forest plots of subgroup analysis.

Supplementary File 4 Sensitivity analysis results.

Supplementary File 5 Funnel plot and publication bias results.
