# Supplementary material for: Global and regional prevalence of multimorbidity in the adult population in community settings: a systematic review and meta-analysis
Source: eClinicalMedicine. 2023 Feb 16;57:101860. doi: 10.1016/j.eclinm.2023.101860 (PMC9971315; doi:10.1016/j.eclinm.2023.101860)
Supplement: Supplementary File 1 — S1. Study quality assessment details for cohort, and cross-sectional studies by New-Castle Ottawa Scale. [file mmc1.docx]

**Supplementary File 1**

**Quality Assessment of Included Studies by New-Castle Ottawa Scale (Adapted)**

| **Cross-sectional Study** | | | | | | | | |
| --- | --- | --- | --- | --- | --- | --- | --- | --- |
| **Authors** | **Year of Publication** | **Country** | **Journal** | **Selection- no. of star (★)** | **Comparability- no. of star (★)** | **Outcome - no. of star (★)** | **Total Score** | **Overall Risk of Bias** |
| Ba et al. | 2019 | Vietnam | Journal of Comorbidity | 4 | 2 | 2 | 8 | Low |
| Banjare et al. | 2014 | India | PLoS ONE | 3 | 2 | 2 | 7 | Moderate |
| Tucker-Seeley et al. | 2011 | USA | BMC Public Health | 5 | 1 | 2 | 8 | Low |
| Chang et al. | 2019 | South Africa | BMJ Global Health | 5 | 0 | 2 | 7 | Moderate |
| Alimohammadian et al. | 2017 | Iran | BMJ Open | 4 | 2 | 2 | 8 | Low |
| Stanley et al. | 2018 | New Zealand | BMJ Open | 5 | 2 | 3 | 10 | Low |
| Agborsangaya et al. | 2012 | Canada | BMC Public Health | 4 | 1 | 2 | 7 | Moderate |
| Kunna et al. | 2017 | China | International Journal for Equity in Health | 5 | 2 | 2 | 9 | Low |
| Kunna et al. | 2017 | Ghana | International Journal for Equity in Health | 5 | 2 | 2 | 9 | Low |
| Araujo et al. | 2018 | Brazil | BMJ Open | 5 | 1 | 2 | 8 | Low |
| Ha et al. | 2015 | Vietnam | International Journal for Equity in Health | 5 | 1 | 2 | 8 | Low |
| Alaba et al. | 2013 | South Africa | International Journal for Equity in Health | 4 | 1 | 2 | 7 | Moderate |
| Puth et al. | 2017 | Germany | BMC Public Health | 3 | 2 | 2 | 7 | Moderate |
| Nunes et al. | 2017 | Brazil | BMJ Open | 5 | 2 | 2 | 9 | Low |
| Phaswana-Mafuya et al. | 2013 | South Africa | Global Health Action | 2 | 1 | 2 | 5 | High |
| Singh et al. | 2018 | South Asia | Journal of Public Health | 5 | 1 | 2 | 8 | Low |
| Frolich et al. | 2019 | Denmark | PLoS ONE | 5 | 1 | 2 | 8 | Low |
| Khan et al. | 2019 | Bangladesh | BMJ Open | 4 | 1 | 2 | 7 | Moderate |
| Roberts et al. | 2015 | Canada | Health Promotion and Chronic Disease Prevention in Canada | 4 | 1 | 2 | 7 | Moderate |
| Pache et al. | 2015 | Switzerland | BMC Public Health | 5 | 2 | 3 | 10 | Low |
| Nunes et al. | 2015 | Brazil | BMC Public Health | 4 | 1 | 2 | 7 | Moderate |
| Nunes et al. | 2016 | Brazil | International Journal of Public Health | 3 | 0 | 2 | 5 | High |
| Wu et al. | 2013 | China | PLoS ONE | 3 | 2 | 2 | 7 | Moderate |
| Mahwati et al. | 2014 | Indonesia | National Public Health Journal | 4 | 1 | 2 | 7 | Moderate |
| Ramond-Roquin et al. | 2016 | Canada | BioMed Research International | 3 | 1 | 2 | 6 | Moderate |
| Khanam et al. | 2011 | Bangladesh | Journal of Health, Population and Nutrition | 4 | 2 | 3 | 9 | Low |
| Nunes et al. | 2018 | Brazil | Rev Saude Publica | 4 | 2 | 2 | 8 | Low |
| Vargese et al. | 2019 | India | Clinical Epidemiology and Global Health | 4 | 2 | 1 | 7 | Moderate |
| Orueta et al. | 2013 | Spain | BMC Public Health | 5 | 0 | 3 | 8 | Low |
| Mondor et al. | 2018 | Canada | International Journal for Equity in Health | 5 | 0 | 3 | 8 | Low |
| Jerliu et al. | 2013 | Kosovo | BMC Geriatrics | 4 | 1 | 2 | 7 | Moderate |
| Picco et al. | 2016 | Singapore | BMC Health Services Research | 4 | 2 | 2 | 8 | Low |
| Kirchberger et al. | 2012 | Germany | PLoS ONE | 4 | 2 | 2 | 8 | Low |
| Taylor et al. | 2010 | Australia | BMC Public Health | 4 | 2 | 2 | 8 | Low |
| Hu et al. | 2019 | Taiwan | BMJ Open | 5 | 0 | 2 | 7 | Moderate |
| Li et al. | 2019 | China | Journal of Public Health | 5 | 2 | 2 | 9 | Low |
| Dung et al. | 2016 | Vietnam | International Journal on Ageing in Developing Countries | 4 | 2 | 1 | 7 | Moderate |
| Costa et al. | 2018 | Brazil | Reports in Public Health | 5 | 0 | 2 | 7 | Moderate |
| Hussain et al. | 2015 | Indonesia | BMJ Open | 5 | 2 | 2 | 9 | Low |
| Chung et al. | 2015 | Hong Kong | PLoS ONE | 4 | 2 | 2 | 8 | Low |
| Jovic et al. | 2016 | Serbia | PLoS ONE | 4 | 0 | 2 | 6 | Moderate |
| Kiliari et al. | 2013 | Cyprus | Journal of the Royal Society of Medicine Open | 2 | 0 | 1 | 3 | High |
| Chen et al. | 2018 | China | Geriatrics and Gerontology International | 4 | 2 | 2 | 8 | Low |
| Machado et al. | 2012 | Brazil | Menopause: The Journal of The North American Menopause Society | 3 | 2 | 2 | 7 | Moderate |
| Lenzi et al. | 2016 | Italy | BMJ Open | 5 | 0 | 2 | 7 | Moderate |
| Mini et al. | 2017 | India | BMJ Open | 5 | 2 | 2 | 9 | Low |
| Ge et al. | 2018 | Singapore | PLoS ONE | 5 | 1 | 3 | 9 | Low |
| Park et al. | 2019 | Korea | PLoS ONE | 5 | 1 | 2 | 8 | Low |
| Craig et al. | 2020 | Jamaica | PLoS ONE | 5 | 1 | 2 | 8 | Low |
| Cossec et al. | 2016 | France | The Journal of Nutrition, Health, and Aging | 4 | 2 | 2 | 8 | Low |
| Loza et al. | 2009 | Spain | Seminars in Arthritis and Rheumatism | 5 | 2 | 2 | 9 | Low |
| Marengoni et al. | 2016 | Sweden | European Journal of Internal Medicine | 4 | 1 | 3 | 8 | Low |
| Afshar et al. | 2015 | 28 Countries | BMC Public Health | 4 | 1 | 2 | 7 | Moderate |
| Minh et al. | 2008 | 5 Countries | Preventing Chronic Disease | 5 | 0 | 1 | 6 | Moderate |
| Cheung et al. | 2018 | Hong Kong | BMC Geriatrics | 4 | 1 | 2 | 7 | Moderate |
| Arokiasamy et al. | 2015 | 6 Countries | BMC Medicine | 5 | 2 | 2 | 9 | Low |
| Bao et al. | 2019 | China | Health and Quality of Life Outcomes | 4 | 2 | 2 | 8 | Low |
| Wang et al. | 2015 | China | Public Health | 4 | 1 | 2 | 7 | Moderate |
| Valadares et al. | 2016 | Brazil | Menopause: The Journal of The North American Menopause Society | 3 | 2 | 2 | 7 | Moderate |
| Waterhouse et al. | 2017 | South Africa | International Journal for Equity in Health | 4 | 2 | 2 | 8 | Low |
| Zemedikun et al. | 2018 | UK | Mayo Clinic Proceedings | 5 | 0 | 1 | 6 | Moderate |
| Amaral et al. | 2018 | Brazil | Ciencia and Saude Coletiva | 3 | 2 | 2 | 7 | Moderate |
| Gu et al. | 2016 | China | Archives of Gerontology and Geriatrics | 4 | 2 | 2 | 8 | Low |
| Hien et al. | 2014 | Burkina Faso | Tropical Medicine and International Health | 4 | 2 | 3 | 9 | Low |
| Lawindi et al. | 2018 | Egypt | The Internet Journal of Epidemiology | 4 | 0 | 2 | 6 | Moderate |
| Zhang et al. | 2020 | USA | International Journal of Environmental Research and Public Health | 5 | 2 | 2 | 9 | Low |
| Jankovic et al. | 2017 | Serbia | Public Health | 5 | 2 | 2 | 9 | Low |
| Wang et al. | 2015 | China | PLoS ONE | 4 | 1 | 2 | 7 | Moderate |
| Palladino et al. | 2016 | 16 Countries | Age and Ageing | 5 | 2 | 2 | 9 | Low |
| Lee et al. | 2020 | Korea | International Journal of Environmental Research and Public Health | 5 | 2 | 2 | 9 | Low |
| Aguiar et. al. | 2013 | Brazil | Menopause: The Journal of The North American Menopause Society | 3 | 2 | 2 | 7 | Moderate |
| Vadrevu et al. | 2016 | India | International Journal of Medical Science and Public Health | 4 | 2 | 2 | 8 | Low |
| Camargo-Casas et al. | 2018 | Colombia | Revista De Investigacion Clinica | 4 | 2 | 2 | 8 | Low |
| Laires et al. | 2018 | Portugal | European Journal of Ageing | 5 | 2 | 2 | 9 | Low |
| Wang et al. | 2017 | Australia | Health and Quality of Life Outcomes | 4 | 2 | 2 | 8 | Low |
| Lai et al. | 2019 | Hong Kong | BMJ Open | 5 | 2 | 2 | 9 | Low |
| Fuchs et al. | 2012 | Germany | Bundesgesundheitsblatt | 4 | 0 | 2 | 6 | Moderate |
| Larsen et al. | 2017 | Denmark | PLoS ONE | 4 | 2 | 2 | 8 | Low |
| Islam et al. | 2014 | Australia | PLoS ONE | 5 | 2 | 2 | 9 | Low |
| Ruel et al. | 2014 | Australia | PLoS ONE | 4 | 0 | 1 | 5 | High |
| Su et al. | 2016 | China | BMC Geriatrics | 4 | 1 | 2 | 7 | Moderate |
| Nguyen et al. | 2019 | England | Journal of Aging and Health | 4 | 2 | 2 | 8 | Low |
| Aminisani et al. | 2020 | Iran | BMC Public Health | 4 | 2 | 2 | 8 | Low |
| Hernandez et al. | 2019 | Ireland | Scientific Reports | 3 | 0 | 1 | 4 | High |
| Wister et al. | 2020 | Canada | Journal of Aging and Environment | 5 | 2 | 2 | 9 | Low |
| Zhang et al. | 2019 | China | BMJ Open | 4 | 2 | 2 | 8 | Low |
| Zhao et al. | 2020 | China | Lancet Glob Health | 5 | 2 | 2 | 9 | Low |
| Zhang et al. | 2021 | China | The Journal of Nutrition, Health and Aging | 4 | 2 | 2 | 8 | Low |
| Yao et al. | 2019 | China | Journal of Gerentology: Medical Sciences | 4 | 2 | 2 | 8 | Low |
| Keetile et al. | 2020 | Botswana | PLoS ONE | 4 | 1 | 2 | 7 | Moderate |
| Zou et al. | 2020 | China | Journal of Public Health | 5 | 2 | 2 | 9 | Low |
| de Melo et al. | 2020 | Brazil | Ciênc. saúde coletiva | 3 | 0 | 1 | 4 | High |
| Dhungana et al. | 2021 | Nepal | BMJ Open | 5 | 2 | 3 | 10 | Low |
| Ma et al. | 2020 | China | BMJ Open | 4 | 1 | 2 | 7 | Moderate |
| Kim et al. | 2020 | Korea | Journal of Globe Health | 3 | 2 | 2 | 7 | Moderate |
| Kshatri et al. | 2020 | India | Frontiers in Public Health | 4 | 2 | 2 | 8 | Low |
| Kyprianidou et al. | 2020 | Cyprus | PLoS ONE | 5 | 2 | 2 | 9 | Low |
| Camargo-Casas et al. | 2018 | Colombia | Rev Invest Clin | 4 | 2 | 2 | 8 | Low |
| King et al. | 2019 | USA | American Board of Family Medicine | 5 | 2 | 2 | 9 | Low |
| Bowling et al. | 2019 | USA | Journal of General Internal Medicine | 5 | 1 | 2 | 8 | Low |
| Wilk et al. | 2021 | Canada | Journal of Multimorbidity and Comorbidity | 4 | 2 | 2 | 8 | Low |
| Tomita et al. | 2021 | Tanzania | Age and Ageing | 4 | 2 | 2 | 8 | Low |
| Smith et al. | 2021 | Ireland | Age and Ageing | 4 | 2 | 2 | 8 | Low |
| Delpino et al. | 2021 | Brazil | REV BRAS EPIDEMIOL | 4 | 1 | 2 | 7 | Moderate |
| Marthias et al. | 2021 | Indonesia | BMJ Open | 5 | 2 | 2 | 9 | Low |
| Zhang et al. | 2021 | China | Frontiers in Public Health | 5 | 2 | 2 | 9 | Low |
| Lin et al. | 2021 | Taiwan | Int. J. Environ. Res. Public Health | 4 | 1 | 2 | 7 | Moderate |
| Nicholson et al. | 2021 | Canada | American Geriatrics Society | 4 | 2 | 2 | 8 | Low |
| de Souza et al. | 2021 | 17 Countries | PLoS ONE | 4 | 2 | 2 | 8 | Low |
| Koyanagi et al. | 2021 | 48 Countries | Journal of Alzheimer’s Disease | 4 | 1 | 2 | 7 | Moderate |
| Oh et al. | 2020 | USA | Journal of Racial and Ethnic Health Disparities | 3 | 1 | 2 | 6 | Moderate |
| Shi et al. | 2021 | Brazil | JMIR Public Health Surveillance | 5 | 2 | 2 | 9 | Low |
| Wang et al. | 2021 | China | BMC Public Health | 4 | 1 | 2 | 7 | Moderate |
| Ballesteros et al. | 2021 | Colombia | Scientific Reports | 5 | 2 | 2 | 9 | Low |
| Mohamed et al. | 2021 | Kenya | Global Health | 4 | 2 | 2 | 9 | Low |
| Kanungo et al. | 2021 | India | International Journal of Environmental Research and Public Health | 4 | 0 | 2 | 6 | Moderate |
| Quinaz Romana et al. | 2019 | Portugal | Acta Medica Portuguesa | 4 | 2 | 2 | 8 | Low |
| de Souza et al. | 2019 | Brazil | Reports in Public Health | 4 | 1 | 2 | 7 | Moderate |
| Costa et al. | 2020 | Brazil | Revista de Saude Publica | 5 | 1 | 2 | 8 | Low |
| Keomma et al. | 2021 | Brazil | Revista de Saude Publica | 4 | 2 | 2 | 8 | Low |
| Jürisson et al. | 2021 | Estonia | BMJ Open | 5 | 2 | 2 | 9 | Low |

| **Cohort Study** | | | | | | | | |
| --- | --- | --- | --- | --- | --- | --- | --- | --- |
| **Authors** | **Year of Publication** | **Country** | **Journal** | **Selection- no. of star (★)** | **Comparability- no. of star (★)** | **Outcome - no. of star (★)** | **Total Score** | **Overall Risk of Bias** |
| He et al. | 2021 | China | BMC Public Health | 4 | 2 | 3 | 9 | Low |
| Mounce et al. | 2018 | England | The Annals of Family Medicine | 4 | 0 | 2 | 6 | Moderate |
| Keats et al. | 2017 | Canada | Preventive Medicine | 4 | 2 | 2 | 8 | Low |
| Lujic et al. | 2017 | Australia | PLoS ONE | 4 | 1 | 2 | 7 | Moderate |
| Dhalwani et al. | 2017 | England | Journals of Gerontology: Medical Sciences | 4 | 2 | 2 | 8 | Low |

| **Overall risk of bias** | |
| --- | --- |
| Low | A total point (stars) 8-10 indicates a low risk of bias |
| Moderate | A total point (stars) 6-7 indicates a moderate risk of bias |
| High | A total point (stars) 0-5 indicates a high risk of bias |

**Adapted Newcastle Ottawa Quality Assessment Scale**

**Cross-Sectional Studies [1]**

**Selection (maximum 5)**

**1.** Representativeness of the sample

a. Truly representative of the average in the target population (random sample or whole population) *

b. Somewhat representative of the average in the target population (purposive sampling of representative samples or evidence that the sample is representative of the source population) *

c. Selected group of users/convenience sampling

d. No description of the sampling strategy

**2.** Sample size

a. Justified and satisfactory*

b. Adequately powered to detect a difference (at least 10 events per variable in multivariate analyses)*

c. Not justified

**3.** Non-respondents

a. Comparability between respondents and non-respondents characteristics is established, and the response rate is satisfactory (>80%)*

b. The response rate is unsatisfactory, or the comparability between respondents and non-respondents is unsatisfactory

c. No description of the response rate or the characteristics of the responders & non-responders

**4.** Ascertainment of the exposure (risk factor)

a. Requires some independent validation (in which diagnostic method it was ascertained)**

b. Non-validated measurement*

d. No description

**Comparability (maximum 2)**

**1.** The subjects in different outcome groups are comparable, based on the study design or analysis. Confounding factors are controlled

a. Study controls for the relevant socio-demographic variable: age, gender, education etc.*

b. Study controls for any additional factor *

**Outcome (maximum 3)**

**1.** Assessment of the outcome (Multimorbidity)

a. Requires some independent validation (in which diagnostic method it was ascertained)**

b. Clinical/Hospital/Medical record**

c. Self-report*

d. No description

**2.** Statistical test

a. The statistical test used to analyze the data is clearly described and appropriate, and the measurement of the association is presented as either an OR, CI and P value or a beta coefficient, SE and P value*

b. The statistical test is not appropriate, not described or incomplete

Note: In our scale, we included two stars for the ascertainment of outcome and exposure if the method of determination is described in the study. If any clinical or hospital record is available, then one star is being given. Such studies are observational and performed in hospital mostly.

**Cohort Studies [2, 3]**

Note: A study can be awarded a maximum of one point for each numbered item with the Selection and Outcome categories. A maximum of two points can be given for Comparability.

**Selection (maximum 4)**

**1.** Representativeness of the exposed cohort

a. Truly representative of the average in the target population (random sample or whole)*

b. Somewhat representative of the average in the target population (purposive sampling of representative sample or evidence that the sample is representative of the source population)*

c. Selected group of users/convenient sampling

d. No description of the derivation of the cohort

**2.** Selection of the non-exposed cohort

a. Drawn from the same community as the exposed cohort or hospitals serving the same population as cohort *

b. Drawn from a different source

c. No description of the derivation of the non-exposed cohort

**3.** Ascertainment of exposure (risk factor)

a. Requires some independent validation (in which diagnostic method it was ascertained)*

b. Clinical/Hospital/Medical record*

c. Self-report/structured interview*

d. No description

**4.** Demonstration that outcome of interest (Multimorbidity) was accounted for or not present at start of study

a. Yes *

b. No

**Comparability (maximum 2)**

**1.** Comparability of cohorts on the basis of the design or analysis

a. Study controls for the relevant socio-demographic variable: age, gender, education etc.*

b. Study controls for any additional factor *

**Outcome (maximum 3)**

**1.** Ascertainment of outcome (Multimorbidity)

a. Requires some independent validation (in which diagnostic method it was ascertained)*

b. Clinical/Hospital/Medical record*

c. Self-reported with no reference to primary record

d. No description

**2.** Was follow-up long enough for outcomes to occur?

a. Yes (>1 year)*

b. No

**3.** Adequacy of follow-up of cohorts

a. Complete follow-up – all subjects accounted for *

b. Subjects lost to follow-up unlikely to introduce bias – small number lost (<20%) or attrition described and accounted for in analysis *

c. Follow up rate not adequate and no description of those lost

d. No statement

**Adapted from previous published literature:**

1. Herzog R, Álvarez-Pasquin MJ, Díaz C, Del Barrio JL, Estrada JM, Gil Á. Are healthcare workers’ intentions to vaccinate related to their knowledge, beliefs and attitudes? A systematic review. *BMC Public Health* 2013; **13**: 154.
2. Ottawa Hospital Research Institute. http://www.ohri.ca/programs/clinical_epidemiology/oxford.asp (accessed Oct 23, 2019).
3. Epstein S, Roberts E, Sedgwick R, *et al.* Poor school attendance and exclusion: a systematic review protocol on educational risk factors for self-harm and suicidal behaviours. *BMJ Open* 2018; **8**: e023953.
