# Supplementary material for: Global and regional prevalence of multimorbidity in the adult population in community settings: a systematic review and meta-analysis
Source: eClinicalMedicine. 2023 Feb 16;57:101860. doi: 10.1016/j.eclinm.2023.101860 (PMC9971315; doi:10.1016/j.eclinm.2023.101860)
Supplement: Supplementary File 3 — Forest plots of subgroup analysis. [file mmc3.docx]

Table of Contents

[Forest plot of subgroup: Africa 1](#_Toc117675115)

[Forest plot of subgroup: Europe 3](#_Toc117675116)

[Forest plot of subgroup: North America 4](#_Toc117675117)

[Forest plot of subgroup: Oceania 5](#_Toc117675118)

[Forest plot of subgroup: South America 6](#_Toc117675119)

[Forest plot of subgroup: High income 7](#_Toc117675120)

[Forest plot of subgroup: Upper middle income 8](#_Toc117675121)

[Forest plot of subgroup: Low and lower middle income 9](#_Toc117675122)

[Forest plot of subgroup: Cross section 10](#_Toc117675123)

[Forest plot of subgroup: Cohort 11](#_Toc117675124)

[Forest plot of subgroup: 5-9 conditions 12](#_Toc117675125)

[Forest plot of subgroup: 10-19 conditions 13](#_Toc117675126)

[Forest plot of subgroup: ≥20 conditions 14](#_Toc117675127)

[Forest plot of subgroup: Female 15](#_Toc117675128)

[Forest plot of subgroup: Male 16](#_Toc117675129)

[Forest plot of subgroup: Age ≥30 years 17](#_Toc117675130)

[Forest plot of subgroup: Age ≥40 years 18](#_Toc117675131)

[Forest plot of subgroup: Age ≥50 years 19](#_Toc117675132)

[Forest plot of subgroup: Age ≥60 years 20](#_Toc117675133)

Forest plot of subgroup: Africa ****Forest plot of subgroup: Asia ****

Forest plot of subgroup: Europe ****

Forest plot of subgroup: North America ****

Forest plot of subgroup: Oceania ****

# Forest plot of subgroup: South America

# Forest plot of subgroup: High income

Forest plot of subgroup: Upper middle income ****

Forest plot of subgroup: Low and lower middle income ****

Forest plot of subgroup: Cross section ****

Forest plot of subgroup: Cohort ****

Forest plot of subgroup: 5-9 conditions ****

Forest plot of subgroup: 10-19 conditions ****

Forest plot of subgroup: ≥20 conditions ****

Forest plot of subgroup: Female ****

Forest plot of subgroup: Male ****

Forest plot of subgroup: Age ≥30 years ****

Forest plot of subgroup: Age ≥40 years ****

Forest plot of subgroup: Age ≥50 years ****

Forest plot of subgroup: Age ≥60 years ****

Forest plot of subgroup: Year 2000-2005

Forest plot of subgroup: Year 2006-2010

Forest plot of subgroup: Year 2011-2015

Forest plot of subgroup: Year 2015-2021
