# Supplementary material for: Global and regional prevalence of multimorbidity in the adult population in community settings: a systematic review and meta-analysis
Source: eClinicalMedicine. 2023 Feb 16;57:101860. doi: 10.1016/j.eclinm.2023.101860 (PMC9971315; doi:10.1016/j.eclinm.2023.101860)

Sensitivity analysis

Table of Contents

SENSITIVITY ANALYSIS........ 1

SA1: STUDIES WITH SAMPLE SIZE MORE THAN 1000 2

SA2: STUDIES WITH SAMPLE SIZE MORE THAN 5000 3

SA3: STUDIES WITH SAMPLE SIZE MORE THAN 10000 4

SA4: STUDIES WITH EXCLUDING STUDIES OF AFRICA........ 5

SA5: STUDIES WITH EXCLUDING STUDIES THAT REPORTED PREVALENCE LESS THAN 20% OR ABOVE 80%........ 6

SA6: WITH HIGH-QUALITY PAPERS 6

SA1: Studies with sample size more than 1000

Authors Name, Publication Year	Events/ Study Participants	Proportion [95% CI]
Koyanagi et al.,2021	8544/224842	0.038 [0.037, 0.039]
Alaba et al.,2013	466/11638	0.040 [0.036, 0.044]
Keetile et al.,2020	63/1178	0.053 [0.041, 0.066]
Minh et al.,2008	1332/18494	0.072 [0.068, 0.076]
Afshar et al.,2015	9782/125404	0.078 [0.077, 0.079]
Wilk et al.,2021	8125/100803	0.081 [0.079, 0.082]
Khan et al.,2019	1036/12338	0.084 [0.079, 0.089]
Lai et al.,2019	1536/17396	0.088 [0.084, 0.093]
Singh et al.,2018	1531/16287	0.094 [0.090, 0.098]
Costa et al.,2020	2543/23329	0.109 [0.105, 0.113]
Chung et al.,2015	3227/25780	0.125 [0.121, 0.129]
Waterhouse et al.,2017	394/3055	0.129 [0.117, 0.141]
Roberts et al.,2015	13597/105406	0.129 [0.127, 0.131]
Cossect et al.,2016	586/4236	0.138 [0.128, 0.149]
Dhungana et al.,2021	1247/8931	0.140 [0.132, 0.147]
Lenzi et al.,2016	574208/3759836	0.153 [0.152, 0.153]
Mahwati et al.,2014	468/2960	0.158 [0.145, 0.171]
Zou et al.,2020	81369/512888	0.159 [0.158, 0.160]
Li et al.,2019	776/4833	0.161 [0.150, 0.171]
Ba et al.,2019	276/1680	0.164 [0.147, 0.182]
Taylor et al.,2010	547/3206	0.171 [0.158, 0.184]
Shi et al.,2021	145355/795271	0.183 [0.182, 0.184]
Wu et al.,2013	2487/13157	0.189 [0.182, 0.196]
Agborsangaya et al.,2012	952/5010	0.190 [0.179, 0.201]
Zemedikun et al.,2018	95710/502643	0.190 [0.189, 0.191]
Alimohammadian et al.,2017	9690/49946	0.194 [0.191, 0.197]
Lawindi et al.,2018	454/2317	0.196 [0.180, 0.212]
Bao et al.,2019	3773/18137	0.208 [0.202, 0.214]
Dhalwani et al.,2017	1155/5476	0.211 [0.200, 0.222]
Frolich et al.,2019	301789/1397173	0.216 [0.215, 0.217]
Arokiasamy et al.,2015	9250/42236	0.219 [0.215, 0.223]
Marthias et al.,2021	809/3678	0.220 [0.207, 0.233]
Nunes et al.,2017	13365/60202	0.222 [0.219, 0.225]
Delpino et al.,2021	14674/65803	0.223 [0.220, 0.226]
Phaswana–Mafuya et al.,2013	819/3638	0.225 [0.212, 0.239]
Kim et al.,2020	16187/68590	0.236 [0.233, 0.239]
Craig et al.,2020	615/2551	0.241 [0.224, 0.258]
Wang et al.,2015	5294/21435	0.247 [0.241, 0.253]
Tomita et al.,2021	570/2299	0.248 [0.230, 0.266]
Kyprianidou et al.,2020	293/1140	0.257 [0.232, 0.282]
Jovic et al.,2016	3525/13103	0.269 [0.261, 0.277]
Lin et al.,2021	1039/3739	0.278 [0.264, 0.292]
Stanley et al.,2018	972222/3489747	0.279 [0.278, 0.279]
Mohamed et al.,2021	576/2003	0.288 [0.268, 0.307]
Wang et al.,2017	2539/8820	0.288 [0.278, 0.297]
Araujo et al.,2018	1160/4001	0.290 [0.276, 0.304]
Nunes et al.,2016	852/2927	0.291 [0.275, 0.308]
Loza et al.,2009	650/2192	0.297 [0.277, 0.316]
Kunna et al.,2017	3509/11814	0.297 [0.289, 0.305]
Jankovic et al.,2017	4151/13765	0.302 [0.294, 0.309]
Kunna et al.,2017	1223/4050	0.302 [0.288, 0.316]
Zhang et al.,2021	985/3250	0.303 [0.287, 0.319]
Hu et al.,2019	434576/1429527	0.304 [0.303, 0.305]
Mini et al.,2017	3025/9852	0.307 [0.298, 0.316]
He et al.,2021	15706/50100	0.313 [0.309, 0.318]
Ruel et al.,2014	593/1854	0.320 [0.299, 0.341]
Mondor et al.,2018	9110/27195	0.335 [0.329, 0.341]
de Souza et al.,2021	21458/63844	0.336 [0.332, 0.340]
Mounce et al.,2018	1552/4564	0.340 [0.326, 0.354]
Tucker–Seeley et al.,2011	2586/7305	0.354 [0.343, 0.365]
Hussain et al.,2015	3369/9438	0.357 [0.347, 0.367]
Aminisani et al.,2020	546/1493	0.366 [0.341, 0.390]
Ge et al.,2018	715/1940	0.369 [0.347, 0.390]
Larsen et al.,2017	60045/162283	0.370 [0.368, 0.372]
Palladino et al.,2016	21047/56427	0.373 [0.369, 0.377]
Lujic et al.,2017	33792/90352	0.374 [0.371, 0.377]
Quinaz Romana et al.,2019	1860/4911	0.379 [0.365, 0.392]
Keats et al.,2017	7142/18709	0.382 [0.375, 0.389]
Park et al.,2019	3264/8370	0.390 [0.380, 0.400]
Ha et al.,2015	941/2400	0.392 [0.373, 0.412]
Puth et al. ,2017	7640/19294	0.396 [0.389, 0.403]
Ju..risson et al.,2021	361940/909477	0.398 [0.397, 0.399]
Keomma et al.,2021	408/1019	0.400 [0.370, 0.430]
Fuchs et al.,2012	8526/21262	0.401 [0.394, 0.408]
Camargo–Casas et al.,2018	808/2000	0.404 [0.382, 0.426]
Camargo–Casas et al.,2018	808/2000	0.404 [0.382, 0.426]
Zhang et al.,2020	55411/132666	0.418 [0.415, 0.420]
Cheung et al.,2018	1094/2618	0.418 [0.399, 0.437]
Yao et al.,2019	8413/19841	0.424 [0.417, 0.431]
Zhang et al.,2019	5104/11707	0.436 [0.427, 0.445]
Lee et al.,2020	4922/11232	0.438 [0.429, 0.447]
Dung et al.,2016	1224/2789	0.439 [0.420, 0.457]
Laires et al.,2018	6671/15196	0.439 [0.431, 0.447]
Jerliu et al.,2013	854/1890	0.452 [0.429, 0.474]
Chen et al.,2018	1700/3737	0.455 [0.439, 0.471]
Su et al.,2016	1012/2058	0.492 [0.470, 0.513]
Gu et al.,2016	1211/2452	0.494 [0.474, 0.514]
Smith et al.,2021	2991/5946	0.503 [0.490, 0.516]
Picco et al.,2016	1321/2565	0.515 [0.496, 0.534]
Islam et al.,2014	2378/4574	0.520 [0.505, 0.534]
Marengoni et al.,2016	1654/3155	0.524 [0.507, 0.542]
Laercio et al.,2020	6212/11697	0.531 [0.522, 0.540]
Zhang et al.,2021	977/1837	0.532 [0.509, 0.555]
Oh et al.,2020	2807/5191	0.541 [0.527, 0.554]
Xiaochen et al.,2020	10673/19656	0.543 [0.536, 0.550]
Pache et al.,2015	2091/3714	0.563 [0.547, 0.579]
Kirchberger et al.,2012	2383/4067	0.586 [0.571, 0.601]
Kanungo et al.,2021	35978/59764	0.602 [0.598, 0.606]
King et al.,2019	3342/5541	0.603 [0.590, 0.616]
Ballesteros et al.,2021	10615/17571	0.604 [0.597, 0.611]
Zhao et al.,2020	7320/11817	0.619 [0.611, 0.628]
Ramond–Roquin et al.,2016	1091/1710	0.638 [0.615, 0.661]
Wister et al.,2020	10075/15711	0.641 [0.634, 0.649]
Orueta et al.1,2013	299233/452698	0.661 [0.660, 0.662]
Nunes et al.,2018	6381/9412	0.678 [0.669, 0.687]
Chang et al.,2019	2699/3889	0.694 [0.680, 0.708]
Bowling et al.,2019	3053/4217	0.724 [0.710, 0.737]
Hernandez et al.,2019	4472/6101	0.733 [0.722, 0.744]
Wang et al.,2021	1390/1871	0.743 [0.723, 0.763]
Nicholson et al.,2021	8404/11161	0.753 [0.745, 0.761]
Nguyen et al.,2019	7409/9171	0.808 [0.800, 0.816]
Nunes et al.,2015	1295/1593	0.813 [0.794, 0.832]
Wang et al.,2015	1339/1480	0.905 [0.890, 0.920]
Costa et al.,2018	1347/1451	0.928 [0.915, 0.942]
Summary: Random effects model	3855328/15393995	0.361 [0.337, 0.384]
Heterogeneity I^2 = 98.8%		

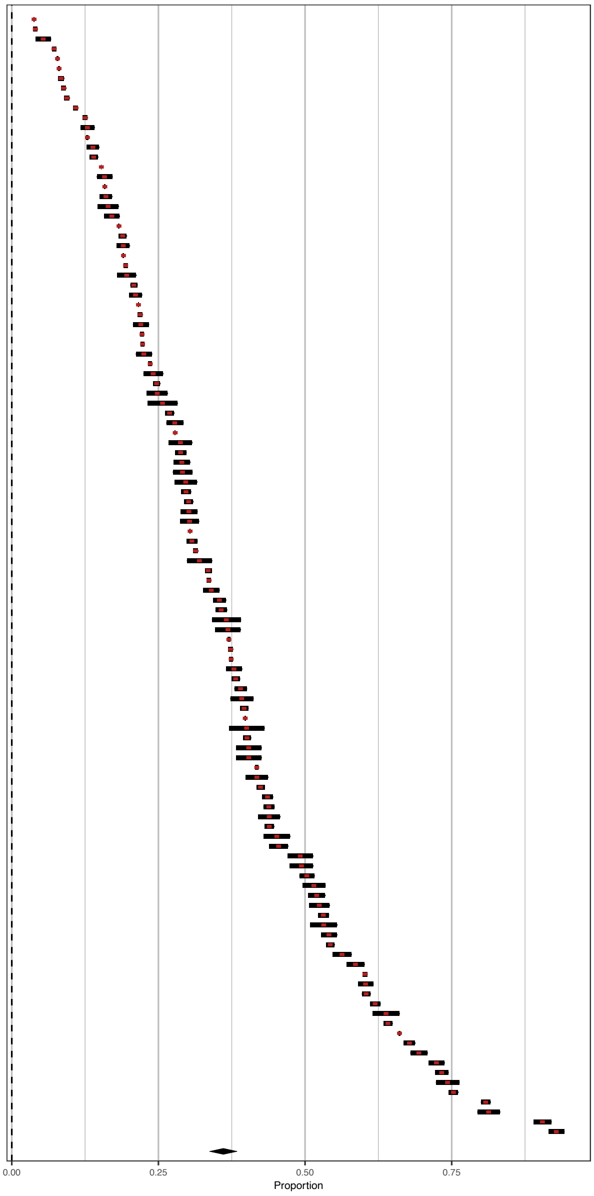

SA2: Studies with sample size more than 5000

Authors Name, Publication Year	Events/ Study Participants	Proportion [95% CI]
Koyanagi et al.,2021	8544/224842	0.038 [0.037, 0.039]
Alaba et al.,2013	466/11638	0.040 [0.036, 0.044]
Minh et al.,2008	1332/18494	0.072 [0.068, 0.076]
Afshar et al.,2015	9782/125404	0.078 [0.077, 0.079]
Wilk et al.,2021	8125/100803	0.081 [0.079, 0.082]
Khan et al.,2019	1036/12338	0.084 [0.079, 0.089]
Lai et al.,2019	1536/17396	0.088 [0.084, 0.093]
Singh et al.,2018	1531/16287	0.094 [0.090, 0.098]
Costa et al.,2020	2543/23329	0.109 [0.105, 0.113]
Chung et al.,2015	3227/25780	0.125 [0.121, 0.129]
Roberts et al.,2015	13597/105406	0.129 [0.127, 0.131]
Dhungana et al.,2021	1247/8931	0.140 [0.132, 0.147]
Lenzi et al.,2016	574208/3759836	0.153 [0.152, 0.153]
Zou et al.,2020	81369/512888	0.159 [0.158, 0.160]
Shi et al.,2021	145355/795271	0.183 [0.182, 0.184]
Wu et al.,2013	2487/13157	0.189 [0.182, 0.196]
Agborsangaya et al.,2012	952/5010	0.190 [0.179, 0.201]
Zemedikun et al.,2018	95710/502643	0.190 [0.189, 0.191]
Alimohammadian et al.,2017	9690/49946	0.194 [0.191, 0.197]
Bao et al.,2019	3773/18137	0.208 [0.202, 0.214]
Dhalwani et al.,2017	1155/5476	0.211 [0.200, 0.222]
Frolich et al.,2019	301789/1397173	0.216 [0.215, 0.217]
Arokiasamy et al.,2015	9250/42236	0.219 [0.215, 0.223]
Nunes et al.,2017	13365/60202	0.222 [0.219, 0.225]
Delpino et al.,2021	14674/65803	0.223 [0.220, 0.226]
Kim et al.,2020	16187/68590	0.236 [0.233, 0.239]
Wang et al.,2015	5294/21435	0.247 [0.241, 0.253]
Jovic et al.,2016	3525/13103	0.269 [0.261, 0.277]
Stanley et al.,2018	972222/3489747	0.279 [0.278, 0.279]
Wang et al.,2017	2539/8820	0.288 [0.278, 0.297]
Kunna et al.,2017	3509/11814	0.297 [0.289, 0.305]
Jankovic et al.,2017	4151/13765	0.302 [0.294, 0.309]
Hu et al.,2019	434576/1429527	0.304 [0.303, 0.305]
Mini et al.,2017	3025/9852	0.307 [0.298, 0.316]
He et al.,2021	15706/50100	0.313 [0.309, 0.318]
Mondor et al.,2018	9110/27195	0.335 [0.329, 0.341]
de Souza et al.,2021	21458/63844	0.336 [0.332, 0.340]
Tucker−Seeley et al.,2011	2586/7305	0.354 [0.343, 0.365]
Hussain et al.,2015	3369/9438	0.357 [0.347, 0.367]
Larsen et al.,2017	60045/162283	0.370 [0.368, 0.372]
Palladino et al.,2016	21047/56427	0.373 [0.369, 0.377]
Lujic et al.,2017	33792/90352	0.374 [0.371, 0.377]
Keats et al.,2017	7142/18709	0.382 [0.375, 0.389]
Park et al.,2019	3264/8370	0.390 [0.380, 0.400]
Puth et al. ,2017	7640/19294	0.396 [0.389, 0.403]
Ju..risson et al.,2021	361940/909477	0.398 [0.397, 0.399]
Fuchs et al.,2012	8526/21262	0.401 [0.394, 0.408]
Zhang et al.,2020	55411/132666	0.418 [0.415, 0.420]
Yao et al.,2019	8413/19841	0.424 [0.417, 0.431]
Zhang et al.,2019	5104/11707	0.436 [0.427, 0.445]
Lee et al.,2020	4922/11232	0.438 [0.429, 0.447]
Laires et al.,2018	6671/15196	0.439 [0.431, 0.447]
Smith et al.,2021	2991/5946	0.503 [0.490, 0.516]
Laercio et al.,2020	6212/11697	0.531 [0.522, 0.540]
Oh et al.,2020	2807/5191	0.541 [0.527, 0.554]
Xiaochen et al.,2020	10673/19656	0.543 [0.536, 0.550]
Kanungo et al.,2021	35978/59764	0.602 [0.598, 0.606]
King et al.,2019	3342/5541	0.603 [0.590, 0.616]
Ballesteros et al.,2021	10615/17571	0.604 [0.597, 0.611]
Zhao et al.,2020	7320/11817	0.619 [0.611, 0.628]
Wister et al.,2020	10075/15711	0.641 [0.634, 0.649]
Orueta et al.1,2013	299233/452698	0.661 [0.660, 0.662]
Nunes et al.,2018	6381/9412	0.678 [0.669, 0.687]
Hernandez et al.,2019	4472/6101	0.733 [0.722, 0.744]
Nicholson et al.,2021	8404/11161	0.753 [0.745, 0.761]
Nguyen et al.,2019	7409/9171	0.808 [0.800, 0.816]
Summary: Random effects model	3803829/15261214	
Heterogeneity I^2 = 98.8%		0.332 [0.302, 0.362]
No. of studies combined:	66	

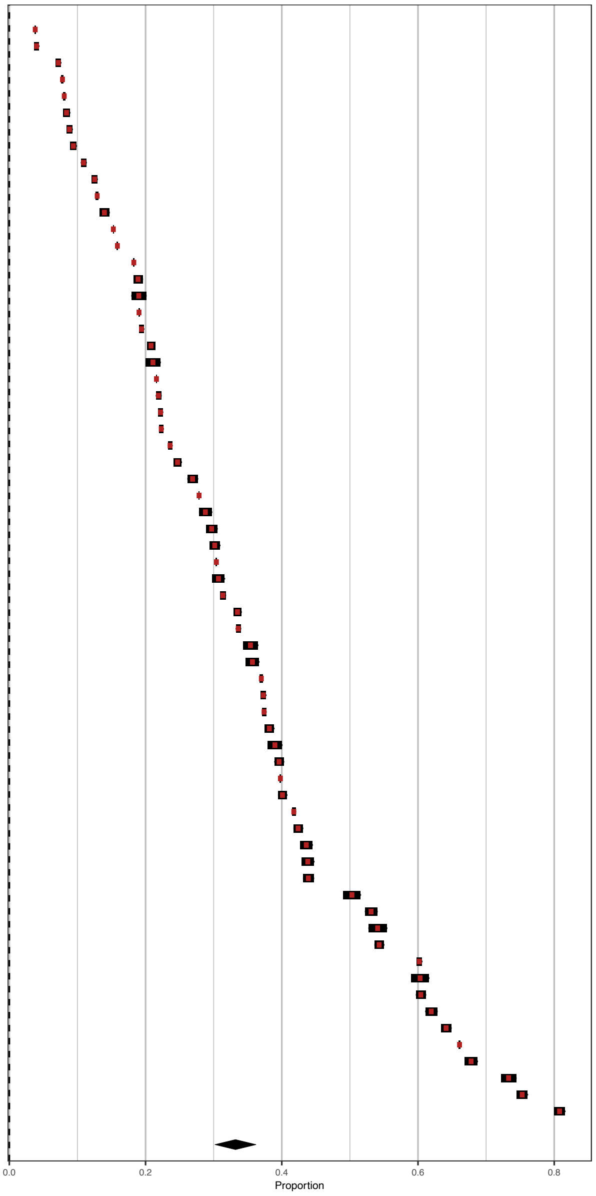

SA3: Studies with sample size more than 10000

Authors Name, Publication Year	Events/ Study Participants	Proportion [95% CI]
Koyanagi et al.,2021	8544/224842	0.038 [0.037, 0.039]
Alaba et al.,2013	466/11638	0.040 [0.036, 0.044]
Minh et al.,2008	1332/18494	0.072 [0.068, 0.076]
Afshar et al.,2015	9782/125404	0.078 [0.077, 0.079]
Wilk et al.,2021	8125/100803	0.081 [0.079, 0.082]
Khan et al.,2019	1036/12338	0.084 [0.079, 0.089]
Lai et al.,2019	1536/17396	0.088 [0.084, 0.093]
Singh et al.,2018	1531/16287	0.094 [0.090, 0.098]
Costa et al.,2020	2543/23329	0.109 [0.105, 0.113]
Chung et al.,2015	3227/25780	0.125 [0.121, 0.129]
Roberts et al.,2015	13597/105406	0.129 [0.127, 0.131]
Lenzi et al.,2016	574208/3759836	0.153 [0.152, 0.153]
Zou et al.,2020	81369/512888	0.159 [0.158, 0.160]
Shi et al.,2021	145355/795271	0.183 [0.182, 0.184]
Wu et al.,2013	2487/13157	0.189 [0.182, 0.196]
Zemedikun et al.,2018	95710/502643	0.190 [0.189, 0.191]
Alimohammadian et al.,2017	9690/49946	0.194 [0.191, 0.197]
Bao et al.,2019	3773/18137	0.208 [0.202, 0.214]
Frolich et al.,2019	301789/1397173	0.216 [0.215, 0.217]
Arokiasamy et al.,2015	9250/42236	0.219 [0.215, 0.223]
Nunes et al.,2017	13365/60202	0.222 [0.219, 0.225]
Delpino et al.,2021	14674/65803	0.223 [0.220, 0.226]
Kim et al.,2020	16187/68590	0.236 [0.233, 0.239]
Wang et al.,2015	5294/21435	0.247 [0.241, 0.253]
Jovic et al.,2016	3525/13103	0.269 [0.261, 0.277]
Stanley et al.,2018	972222/3489747	0.279 [0.278, 0.279]
Kunna et al.,2017	3509/11814	0.297 [0.289, 0.305]
Jankovic et al.,2017	4151/13765	0.302 [0.294, 0.309]
Hu et al.,2019	434576/1429527	0.304 [0.303, 0.305]
He et al.,2021	15706/50100	0.313 [0.309, 0.318]
Mondor et al.,2018	9110/27195	0.335 [0.329, 0.341]
de Souza et al.,2021	21458/63844	0.336 [0.332, 0.340]
Larsen et al.,2017	60045/162283	0.370 [0.368, 0.372]
Palladino et al.,2016	21047/56427	0.373 [0.369, 0.377]
Lujic et al.,2017	33792/90352	0.374 [0.371, 0.377]
Keats et al.,2017	7142/18709	0.382 [0.375, 0.389]
Puth et al. ,2017	7640/19294	0.396 [0.389, 0.403]
Ju..risson et al.,2021	361940/909477	0.398 [0.397, 0.399]
Fuchs et al.,2012	8526/21262	0.401 [0.394, 0.408]
Zhang et al.,2020	55411/132666	0.418 [0.415, 0.420]
Yao et al.,2019	8413/19841	0.424 [0.417, 0.431]
Zhang et al.,2019	5104/11707	0.436 [0.427, 0.445]
Lee et al.,2020	4922/11232	0.438 [0.429, 0.447]
Laires et al.,2018	6671/15196	0.439 [0.431, 0.447]
Laercio et al.,2020	6212/11697	0.531 [0.522, 0.540]
Xiaochen et al.,2020	10673/19656	0.543 [0.536, 0.550]
Kanungo et al.,2021	35978/59764	0.602 [0.598, 0.606]
Ballesteros et al.,2021	10615/17571	0.604 [0.597, 0.611]
Zhao et al.,2020	7320/11817	0.619 [0.611, 0.628]
Wister et al.,2020	10075/15711	0.641 [0.634, 0.649]
Orueta et al.1,2013	299233/452698	0.661 [0.660, 0.662]
Nicholson et al.,2021	8404/11161	0.753 [0.745, 0.761]
Summary: Random effects model	3758290/15156650	
Heterogeneity I^2 = 98.8%		0.304 [0.271, 0.337]
No. of studies combined:	52	

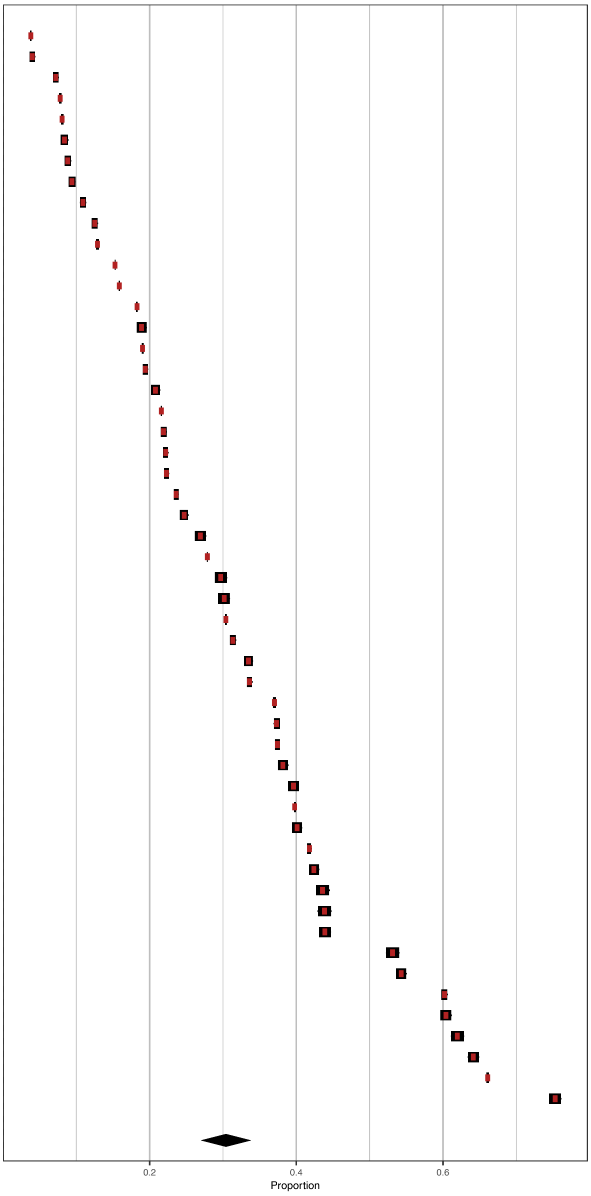

SA4: Studies with excluding studies of Africa

Authors Name, Publication Year	Events/ Study Participants	Proportion [95% CI]
Koyanagi et al.,2021	8544/224842	0.038 [0.037, 0.039]
Minh et al.,2008	1332/18494	0.072 [0.068, 0.076]
Afshar et al.,2015	9782/125404	0.078 [0.077, 0.079]
Wilk et al.,2021	8125/100803	0.081 [0.079, 0.082]
Khan et al.,2019	1036/12338	0.084 [0.079, 0.089]
Lai et al.,2019	1536/17396	0.088 [0.084, 0.093]
Singh et al.,2018	1531/16287	0.094 [0.090, 0.098]
Costa et al.,2020	2543/23329	0.109 [0.105, 0.113]
Chung et al.,2015	3227/25780	0.125 [0.121, 0.129]
Roberts et al.,2015	13597/105406	0.129 [0.127, 0.131]
Cossect et al.,2016	586/4236	0.138 [0.128, 0.149]
Dhungana et al.,2021	1247/8931	0.140 [0.132, 0.147]
Lenzi et al.,2016	574208/3759836	0.153 [0.152, 0.153]
Mahwati et al.,2014	468/2960	0.158 [0.145, 0.171]
Zou et al.,2020	81369/512888	0.159 [0.158, 0.160]
Li et al.,2019	776/4833	0.161 [0.150, 0.171]
Vargese et al.,2019	85/525	0.162 [0.130, 0.193]
Ba et al.,2019	276/1680	0.164 [0.147, 0.182]
Taylor et al.,2010	547/3206	0.171 [0.158, 0.184]
Shi et al.,2021	145355/795271	0.183 [0.182, 0.184]
Wu et al.,2013	2487/13157	0.189 [0.182, 0.196]
Agborsangaya et al.,2012	952/5010	0.190 [0.179, 0.201]
Zemedikun et al.,2018	95710/502643	0.190 [0.189, 0.191]
Alimohammadian et al.,2017	9690/49946	0.194 [0.191, 0.197]
Bao et al.,2019	3773/18137	0.208 [0.202, 0.214]
Dhalwani et al.,2017	1155/5476	0.211 [0.200, 0.222]
Frolich et al.,2019	301789/1397173	0.216 [0.215, 0.217]
Arokiasamy et al.,2015	9250/42236	0.219 [0.215, 0.223]
Marthias et al.,2021	809/3678	0.220 [0.207, 0.233]
Nunes et al.,2017	13365/60202	0.222 [0.219, 0.225]
Delpino et al.,2021	14674/65803	0.223 [0.220, 0.226]
Kim et al.,2020	16187/68590	0.236 [0.233, 0.239]
Craig et al.,2020	615/2551	0.241 [0.224, 0.258]
Wang et al.,2015	5294/21435	0.247 [0.241, 0.253]
Kyprianidou et al.,2020	293/1140	0.257 [0.232, 0.282]
Jovic et al.,2016	3525/13103	0.269 [0.261, 0.277]
Lin et al.,2021	1039/3739	0.278 [0.264, 0.292]
Stanley et al.,2018	972222/3489747	0.279 [0.278, 0.279]
Kiliari et al.,2013	133/465	0.286 [0.245, 0.327]
Wang et al.,2017	2539/8820	0.288 [0.278, 0.297]
Araujo et al.,2018	1160/4001	0.290 [0.276, 0.304]
Nunes et al.,2016	852/2927	0.291 [0.275, 0.308]
Loza et al.,2009	650/2192	0.297 [0.277, 0.316]
Kunna et al.,2017	3509/11814	0.297 [0.289, 0.305]
Jankovic et al.,2017	4151/13765	0.302 [0.294, 0.309]
Zhang et al.,2021	985/3250	0.303 [0.287, 0.319]
Hu et al.,2019	434576/1429527	0.304 [0.303, 0.305]
Mini et al.,2017	3025/9852	0.307 [0.298, 0.316]
He et al.,2021	15706/50100	0.313 [0.309, 0.318]
Ruel et al.,2014	593/1854	0.320 [0.299, 0.341]
Mondor et al.,2018	9110/27195	0.335 [0.329, 0.341]
de Souza et al.,2021	21458/63844	0.336 [0.332, 0.340]
Mounce et al.,2018	1552/4564	0.340 [0.326, 0.354]
Tucker–Seeley et al.,2011	2586/7305	0.354 [0.343, 0.365]
Hussain et al.,2015	3369/9438	0.357 [0.347, 0.367]
Aminisani et al.,2020	546/1493	0.366 [0.341, 0.390]
Ge et al.,2018	715/1940	0.369 [0.347, 0.390]
Larsen et al.,2017	60045/162283	0.370 [0.368, 0.372]
Palladino et al.,2016	21047/56427	0.373 [0.369, 0.377]
Lujic et al.,2017	33792/90352	0.374 [0.371, 0.377]
Quinaz Romana et al.,2019	1860/4911	0.379 [0.365, 0.392]
Keats et al.,2017	7142/18709	0.382 [0.375, 0.389]
Park et al.,2019	3264/8370	0.390 [0.380, 0.400]
Ha et al.,2015	941/2400	0.392 [0.373, 0.412]
de Souza Santos Machado et al.,2012	148/377	0.393 [0.343, 0.442]
Puth et al. ,2017	7640/19294	0.396 [0.389, 0.403]
Ju..risson et al.,2021	361940/909477	0.398 [0.397, 0.399]
Keomma et al.,2021	408/1019	0.400 [0.370, 0.430]
Fuchs et al.,2012	8526/21262	0.401 [0.394, 0.408]
Camargo–Casas et al.,2018	808/2000	0.404 [0.382, 0.426]
Camargo–Casas et al.,2018	808/2000	0.404 [0.382, 0.426]
Zhang et al.,2020	55411/132666	0.418 [0.415, 0.420]
Cheung et al.,2018	1094/2618	0.418 [0.399, 0.437]
Yao et al.,2019	8413/19841	0.424 [0.417, 0.431]
Zhang et al.,2019	5104/11707	0.436 [0.427, 0.445]
Lee et al.,2020	4922/11232	0.438 [0.429, 0.447]
Dung et al.,2016	1224/2789	0.439 [0.420, 0.457]
Laires et al.,2018	6671/15196	0.439 [0.431, 0.447]
Vadrevu et al.,2016	359/815	0.440 [0.406, 0.475]
Jerliu et al.,2013	854/1890	0.452 [0.429, 0.474]
Chen et al.,2018	1700/3737	0.455 [0.439, 0.471]
de Souza et al.,2019	334/733	0.456 [0.420, 0.492]
Kshatri et al.,2020	354/725	0.488 [0.452, 0.525]
Su et al.,2016	1012/2058	0.492 [0.470, 0.513]
Gu et al.,2016	1211/2452	0.494 [0.474, 0.514]
Smith et al.,2021	2991/5946	0.503 [0.490, 0.516]
Picco et al.,2016	1321/2565	0.515 [0.496, 0.534]
Islam et al.,2014	2378/4574	0.520 [0.505, 0.534]
Valadares et al.,2016	391/749	0.522 [0.486, 0.558]
Marengoni et al.,2016	1654/3155	0.524 [0.507, 0.542]
Laercio et al.,2020	6212/11697	0.531 [0.522, 0.540]
Zhang et al.,2021	977/1837	0.532 [0.509, 0.555]
Khanam et al.,2011	243/452	0.538 [0.492, 0.584]
Oh et al.,2020	2807/5191	0.541 [0.527, 0.554]
Xiaochen et al.,2020	10673/19656	0.543 [0.536, 0.550]
Pache et al.,2015	2091/3714	0.563 [0.547, 0.579]
Banjare et al.,2014	176/310	0.568 [0.513, 0.623]
Aguiar et. al.,2013	362/622	0.582 [0.543, 0.621]
Kirchberger et al.,2012	2383/4067	0.586 [0.571, 0.601]
Kanungo et al.,2021	35978/59764	0.602 [0.598, 0.606]
King et al.,2019	3342/5541	0.603 [0.590, 0.616]
Ballesteros et al.,2021	10615/17571	0.604 [0.597, 0.611]
Zhao et al.,2020	7320/11817	0.619 [0.611, 0.628]
Ramond–Roquin et al.,2016	1091/1710	0.638 [0.615, 0.661]
Wister et al.,2020	10075/15711	0.641 [0.634, 0.649]
Orueta et al.1,2013	299233/452698	0.661 [0.660, 0.662]
Amaral et al.,2018	175/264	0.663 [0.606, 0.720]
Nunes et al.,2018	6381/9412	0.678 [0.669, 0.687]
Bowling et al.,2019	3053/4217	0.724 [0.710, 0.737]
Hernandez et al.,2019	4472/6101	0.733 [0.722, 0.744]
Wang et al.,2021	1390/1871	0.743 [0.723, 0.763]
Nicholson et al.,2021	8404/11161	0.753 [0.745, 0.761]
Nguyen et al.,2019	7409/9171	0.808 [0.800, 0.816]
Nunes et al.,2015	1295/1593	0.813 [0.794, 0.832]
Wang et al.,2015	1339/1480	0.905 [0.890, 0.920]
Costa et al.,2018	1347/1451	0.928 [0.915, 0.942]
Summary: Random effects model	3850824/15365965	0.379 [0.356, 0.402]

Heterogeneity I^2 = 98.8%

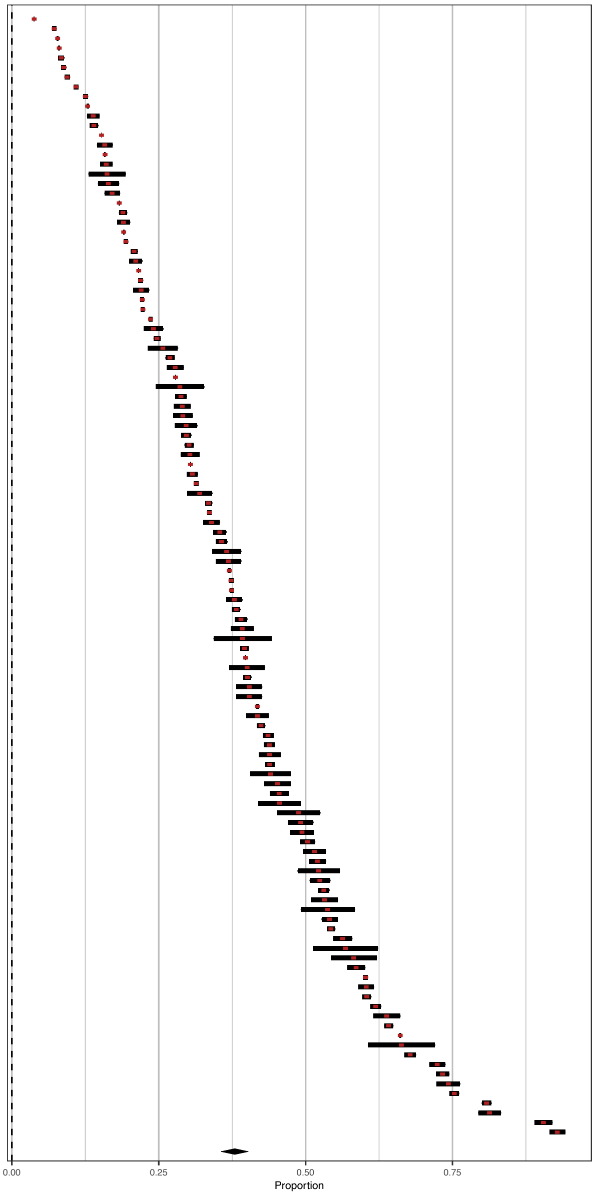

SA5: Studies with excluding studies that reported prevalence less than 20% or above 80%

Authors Name, Publication Year	Events/ Study Participants	Proportion [95% CI]
Bao et al.,2019	3773/18137	0.208 [0.202, 0.214]
Dhalwani et al.,2017	1155/5476	0.211 [0.200, 0.222]
Frolich et al.,2019	301789/1397173	0.216 [0.215, 0.217]
Arokiasamy et al.,2015	9250/42236	0.219 [0.215, 0.223]
Marthias et al.,2021	809/3678	0.220 [0.207, 0.233]
Nunes et al.,2017	13365/60202	0.222 [0.219, 0.225]
Delpino et al.,2021	14674/65803	0.223 [0.220, 0.226]
Phaswana–Mafuya et al.,2013	819/3638	0.225 [0.212, 0.239]
Kim et al.,2020	16187/68590	0.236 [0.233, 0.239]
Craig et al.,2020	615/2551	0.241 [0.224, 0.258]
Wang et al.,2015	5294/21435	0.247 [0.241, 0.253]
Tomita et al.,2021	570/2299	0.248 [0.230, 0.266]
Kyprianidou et al.,2020	293/1140	0.257 [0.232, 0.282]
Jovic et al.,2016	3525/13103	0.269 [0.261, 0.277]
Lin et al.,2021	1039/3739	0.278 [0.264, 0.292]
Stanley et al.,2018	972222/3489747	0.279 [0.278, 0.279]
Kiliari et al.,2013	133/465	0.286 [0.245, 0.327]
Mohamed et al.,2021	576/2003	0.288 [0.268, 0.307]
Wang et al.,2017	2539/8820	0.288 [0.278, 0.297]
Araujo et al.,2018	1160/4001	0.290 [0.276, 0.304]
Nunes et al.,2016	852/2927	0.291 [0.275, 0.308]
Loza et al.,2009	650/2192	0.297 [0.277, 0.316]
Kunna et al.,2017	3509/11814	0.297 [0.289, 0.305]
Jankovic et al.,2017	4151/13765	0.302 [0.294, 0.309]
Kunna et al.,2017	1223/4050	0.302 [0.288, 0.316]
Zhang et al.,2021	985/3250	0.303 [0.287, 0.319]
Hu et al.,2019	434576/1429527	0.304 [0.303, 0.305]
Mini et al.,2017	3025/9852	0.307 [0.298, 0.316]
He et al.,2021	15706/50100	0.313 [0.309, 0.318]
Ruel et al.,2014	593/1854	0.320 [0.299, 0.341]
Mondor et al.,2018	9110/27195	0.335 [0.329, 0.341]
de Souza et al.,2021	21458/63844	0.336 [0.332, 0.340]
Mounce et al.,2018	1552/4564	0.340 [0.326, 0.354]
Tucker–Seeley et al.,2011	2586/7305	0.354 [0.343, 0.365]
Hussain et al.,2015	3369/9438	0.357 [0.347, 0.367]
Aminisani et al.,2020	546/1493	0.366 [0.341, 0.390]
Ge et al.,2018	715/1940	0.369 [0.347, 0.390]
Larsen et al.,2017	60045/162283	0.370 [0.368, 0.372]
Palladino et al.,2016	21047/56427	0.373 [0.369, 0.377]
Lujic et al.,2017	33792/90352	0.374 [0.371, 0.377]
Quinaz Romana et al.,2019	1860/4911	0.379 [0.365, 0.392]
Keats et al.,2017	7142/18709	0.382 [0.375, 0.389]
Park et al.,2019	3264/8370	0.390 [0.380, 0.400]
Ha et al.,2015	941/2400	0.392 [0.373, 0.412]
de Souza Santos Machado et al.,2012	148/377	0.393 [0.343, 0.442]
Puth et al. ,2017	7640/19294	0.396 [0.389, 0.403]
Ju..risson et al.,2021	361940/909477	0.398 [0.397, 0.399]
Keomma et al.,2021	408/1019	0.400 [0.370, 0.430]
Fuchs et al.,2012	8526/21262	0.401 [0.394, 0.408]
Camargo–Casas et al.,2018	808/2000	0.404 [0.382, 0.426]
Camargo–Casas et al.,2018	808/2000	0.404 [0.382, 0.426]
Zhang et al.,2020	55411/132666	0.418 [0.415, 0.420]
Cheung et al.,2018	1094/2618	0.418 [0.399, 0.437]
Yao et al.,2019	8413/19841	0.424 [0.417, 0.431]
Zhang et al.,2019	5104/11707	0.436 [0.427, 0.445]
Lee et al.,2020	4922/11232	0.438 [0.429, 0.447]
Dung et al.,2016	1224/2789	0.439 [0.420, 0.457]
Laires et al.,2018	6671/15196	0.439 [0.431, 0.447]
Vadrevu et al.,2016	359/815	0.440 [0.406, 0.475]
Jerliu et al.,2013	854/1890	0.452 [0.429, 0.474]
Chen et al.,2018	1700/3737	0.455 [0.439, 0.471]
de Souza et al.,2019	334/733	0.456 [0.420, 0.492]
Kshatri et al.,2020	354/725	0.488 [0.452, 0.525]
Su et al.,2016	1012/2058	0.492 [0.470, 0.513]
Gu et al.,2016	1211/2452	0.494 [0.474, 0.514]
Smith et al.,2021	2991/5946	0.503 [0.490, 0.516]
Picco et al.,2016	1321/2565	0.515 [0.496, 0.534]
Islam et al.,2014	2378/4574	0.520 [0.505, 0.534]
Valadares et al.,2016	391/749	0.522 [0.486, 0.558]
Marengoni et al.,2016	1654/3155	0.524 [0.507, 0.542]
Laercio et al.,2020	6212/11697	0.531 [0.522, 0.540]
Zhang et al.,2021	977/1837	0.532 [0.509, 0.555]
Khanam et al.,2011	243/452	0.538 [0.492, 0.584]
Oh et al.,2020	2807/5191	0.541 [0.527, 0.554]
Xiaochen et al.,2020	10673/19656	0.543 [0.536, 0.550]
Pache et al.,2015	2091/3714	0.563 [0.547, 0.579]
Banjare et al.,2014	176/310	0.568 [0.513, 0.623]
Aguiar et. al.,2013	362/622	0.582 [0.543, 0.621]
Kirchberger et al.,2012	2383/4067	0.586 [0.571, 0.601]
Kanungo et al.,2021	35978/59764	0.602 [0.598, 0.606]
King et al.,2019	3342/5541	0.603 [0.590, 0.616]
Ballesteros et al.,2021	10615/17571	0.604 [0.597, 0.611]
Zhao et al.,2020	7320/11817	0.619 [0.611, 0.628]
Ramond–Roquin et al.,2016	1091/1710	0.638 [0.615, 0.661]
Wister et al.,2020	10075/15711	0.641 [0.634, 0.649]
Hien et al.,2014	252/389	0.648 [0.600, 0.695]
Orueta et al.1,2013	299233/452698	0.661 [0.660, 0.662]
Amaral et al.,2018	175/264	0.663 [0.606, 0.720]
Nunes et al.,2018	6381/9412	0.678 [0.669, 0.687]
Chang et al.,2019	2699/3889	0.694 [0.680, 0.708]
Bowling et al.,2019	3053/4217	0.724 [0.710, 0.737]
Hernandez et al.,2019	4472/6101	0.733 [0.722, 0.744]
Wang et al.,2021	1390/1871	0.743 [0.723, 0.763]
Nicholson et al.,2021	8404/11161	0.753 [0.745, 0.761]
Summary: Random effects model	2880564/9033337	0.423 [0.398, 0.447]
Heterogeneity I^2 = 98.8%		

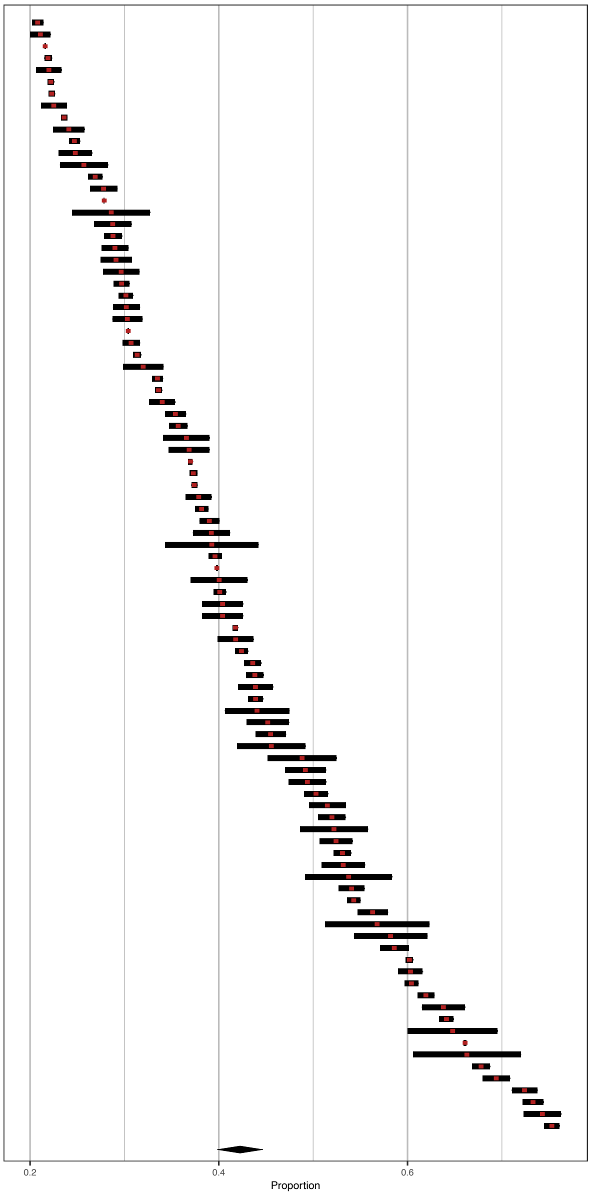

SA6: With high-quality papers

Authors Name, Publication Year	Events/ Study Participants	Proportion [95% CI]
Bao et al.,2019	3773/18137	0.208 [0.202, 0.214]
Frolich et al.,2019	301789/1397173	0.216 [0.215, 0.217]
Arokiasamy et al.,2015	9250/42236	0.219 [0.215, 0.223]
Nunes et al.,2017	13365/60202	0.222 [0.219, 0.225]
Delpino et al.,2021	14674/65803	0.223 [0.220, 0.226]
Kim et al.,2020	16187/68590	0.236 [0.233, 0.239]
Wang et al.,2015	5294/21435	0.247 [0.241, 0.253]
Jovic et al.,2016	3525/13103	0.269 [0.261, 0.277]
Stanley et al.,2018	972222/3489747	0.279 [0.278, 0.279]
Wang et al.,2017	2539/8820	0.288 [0.278, 0.297]
Kunna et al.,2017	3509/11814	0.297 [0.289, 0.305]
Jankovic et al.,2017	4151/13765	0.302 [0.294, 0.309]
Hu et al.,2019	434576/1429527	0.304 [0.303, 0.305]
Mini et al.,2017	3025/9852	0.307 [0.298, 0.316]
He et al.,2021	15706/50100	0.313 [0.309, 0.318]
Mondor et al.,2018	9110/27195	0.335 [0.329, 0.341]
de Souza et al.,2021	21458/63844	0.336 [0.332, 0.340]
Tucker–Seeley et al.,2011	2586/7305	0.354 [0.343, 0.365]
Hussain et al.,2015	3369/9438	0.357 [0.347, 0.367]
Larsen et al.,2017	60045/162283	0.370 [0.368, 0.372]
Palladino et al.,2016	21047/56427	0.373 [0.369, 0.377]
Lujic et al.,2017	33792/90352	0.374 [0.371, 0.377]
Keats et al.,2017	7142/18709	0.382 [0.375, 0.389]
Park et al.,2019	3264/8370	0.390 [0.380, 0.400]
Puth et al. ,2017	7640/19294	0.396 [0.389, 0.403]
Ju..risson et al.,2021	361940/909477	0.398 [0.397, 0.399]
Fuchs et al.,2012	8526/21262	0.401 [0.394, 0.408]
Zhang et al.,2020	55411/132666	0.418 [0.415, 0.420]
Yao et al.,2019	8413/19841	0.424 [0.417, 0.431]
Zhang et al.,2019	5104/11707	0.436 [0.427, 0.445]
Lee et al.,2020	4922/11232	0.438 [0.429, 0.447]
Laires et al.,2018	6671/15196	0.439 [0.431, 0.447]
Laercio et al.,2020	6212/11697	0.531 [0.522, 0.540]
Xiaochen et al.,2020	10673/19656	0.543 [0.536, 0.550]
Kanungo et al.,2021	35978/59764	0.602 [0.598, 0.606]
Ballesteros et al.,2021	10615/17571	0.604 [0.597, 0.611]
Zhao et al.,2020	7320/11817	0.619 [0.611, 0.628]
Wister et al.,2020	10075/15711	0.641 [0.634, 0.649]
Orueta et al.1,2013	299233/452698	0.661 [0.660, 0.662]
Nunes et al.,2018	6381/9412	0.678 [0.669, 0.687]
Nicholson et al.,2021	8404/11161	0.753 [0.745, 0.761]
Summary: Random effects model	2818916/8894389	
Heterogeneity I^2 = 98.8%		0.395 [0.359, 0.430]
No. of studies combined:	41	

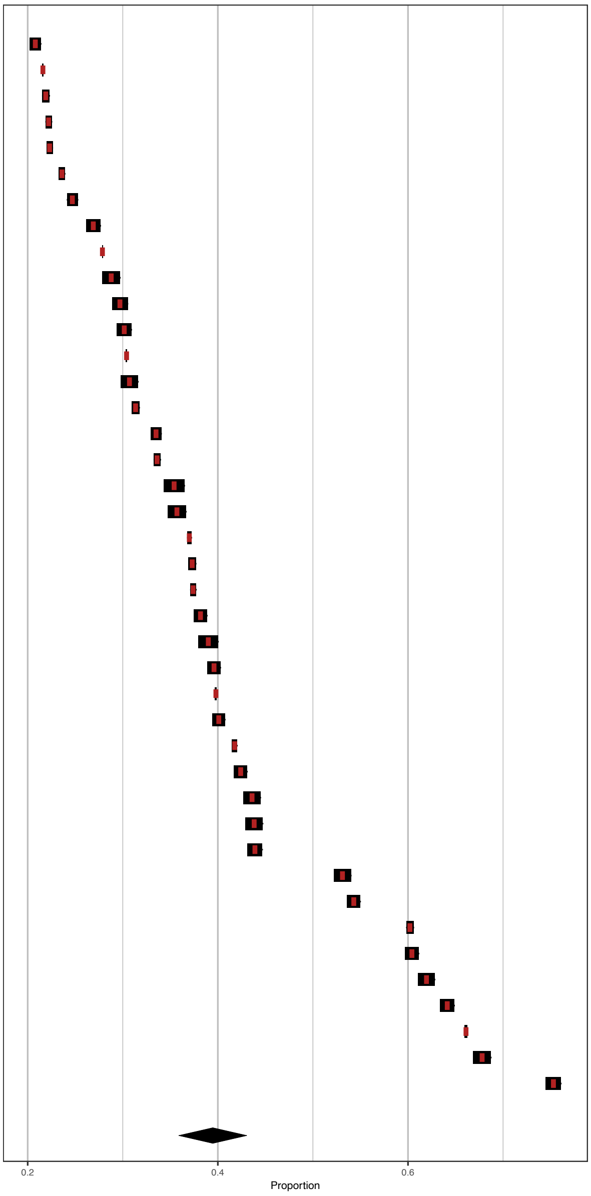

Supplement: Supplementary File 4 — Sensitivity analysis results. [file mmc4.pdf]
