# Supplementary material for: Global and regional prevalence of multimorbidity in the adult population in community settings: a systematic review and meta-analysis
Source: eClinicalMedicine. 2023 Feb 16;57:101860. doi: 10.1016/j.eclinm.2023.101860 (PMC9971315; doi:10.1016/j.eclinm.2023.101860)

Table of Contents

[Funnel plot for investigating association between gender and multimorbidity with 83 studies: 1](#_Toc118049037)

[Funnel plot for proportions with 126 studies: 2](#_Toc118049038)

# Funnel plot for investigating association between gender and multimorbidity with 83 studies:

The funnel plot reveals existence of publication bias though many points fall outside of the 95% confidence region. Each point represents a study; the y-axis represents standard error, and the x-axis displays the ratio of the log odds of the study. However, the egger test indicated there was no statistically significant publication bias (p>0.05).

We also have applied trim-and-fill method to adjust for this publication bias in the analysis. Even though many items fall inside of 95% confidence region, publication bias is nonetheless there.

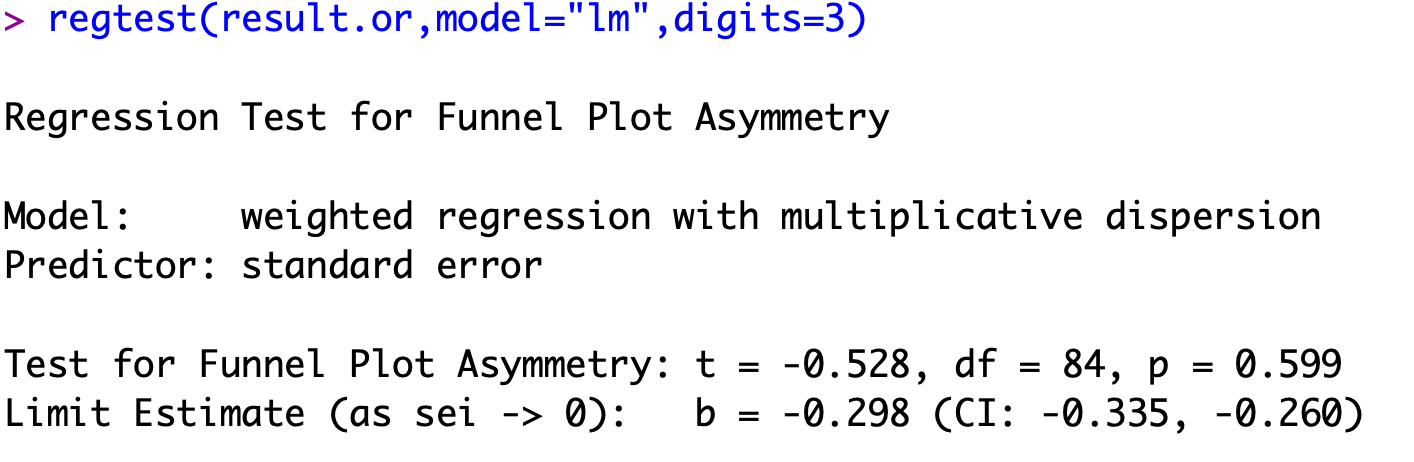


# Funnel plot for proportions with 126 studies:

The funnel plot reveals existence of publication bias though most points fall outside of the 95% confidence region. Each point represents a study; the y-axis represents standard error of the transformed proportions, and the x-axis displays the transformed proportions of the study.

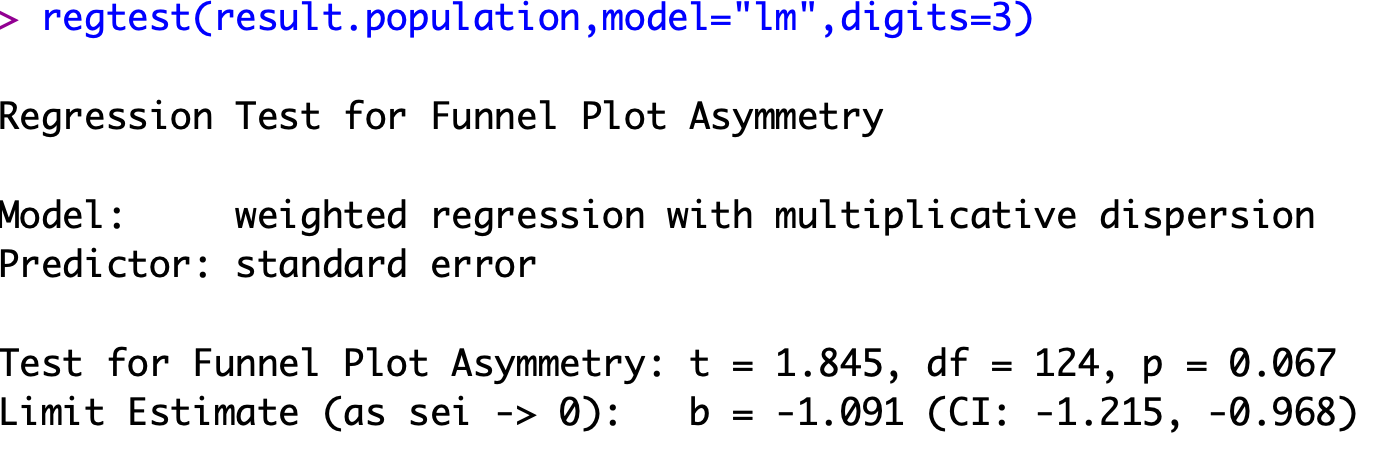

Supplement: Supplementary File 5 — Funnel plot and publication bias results. [file mmc5.docx]
